# Hyaluronic Acid and Controlled Release: A Review

**DOI:** 10.3390/molecules25112649

**Published:** 2020-06-06

**Authors:** Ilker S. Bayer

**Affiliations:** Smart Materials, Istituto Italiano di Tecnologia, Via Morego 30, 16163 Genoa, Italy; ilker.bayer@iit.it; Tel.: +39-388-387-6699; Fax: +39-010-717-81209

**Keywords:** hyaluronan, hyaluronic acid, hydrogel, controlled release, sustained drug delivery, cancer, protein

## Abstract

Hyaluronic acid (HA) also known as hyaluronan, is a natural polysaccharide—an anionic, non-sulfated glycosaminoglycan—commonly found in our bodies. It occurs in the highest concentrations in the eyes and joints. Today HA is used during certain eye surgeries and in the treatment of dry eye disease. It is a remarkable natural lubricant that can be injected into the knee for patients with knee osteoarthritis. HA has also excellent gelling properties due to its capability to bind water very quickly. As such, it is one the most attractive controlled drug release matrices and as such, it is frequently used in various biomedical applications. Due to its reactivity, HA can be cross-linked or conjugated with assorted bio-macromolecules and it can effectively encapsulate several different types of drugs, even at nanoscale. Moreover, the physiological significance of the interactions between HA and its main membrane receptor, CD44 (a cell-surface glycoprotein that modulates cell–cell interactions, cell adhesion and migration), in pathological processes, e.g., cancer, is well recognized and this has resulted in an extensive amount of studies on cancer drug delivery and tumor targeting. HA acts as a therapeutic but also as a tunable matrix for drug release. Thus, this review focuses on controlled or sustained drug release systems assembled from HA and its derivatives. More specifically, recent advances in controlled release of proteins, antiseptics, antibiotics and cancer targeting drugs from HA and its derivatives were reviewed. It was shown that controlled release from HA has many benefits such as optimum drug concentration maintenance, enhanced therapeutic effects, improved efficiency of treatment with less drug, very low or insignificant toxicity and prolonged in vivo release rates.

## 1. Introduction

**Figure 1:**
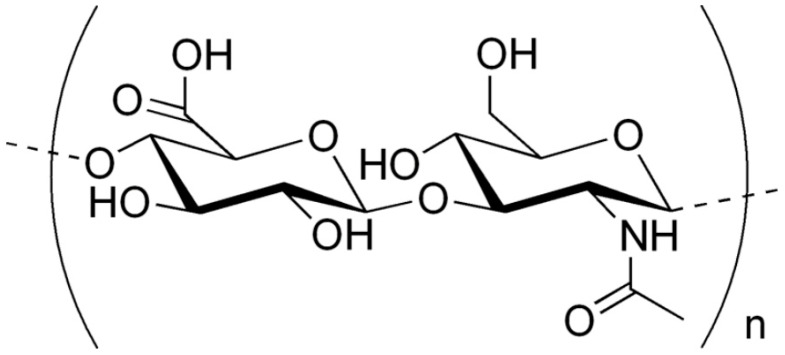
Chemical structure of HA polymer.

Polymers based on natural polysaccharides and their derivatives have significant implications in the development of new generation medical materials and drug delivery systems. Among them, the most common glycosaminoglycan found in vertebrate tissues that is responsible for several vital activities is known as hyaluronic acid (HA) or hyaluronan. HA is a linear glycosaminoglycan (see Figure 1) made up of repeating units of N-acetyl-d-glucosamine and d-glucuronic acid with the monosaccharide units linked together via alternating β-1,3 and β-1,4 glycosidic bonds [1,2]. Its chemical structure is substantially consistent, with the exception of occasional deacetylated glucosamine residues [2]. In physiological conditions, HA takes the form of a sodium salt that is negatively charged and highly hydrophilic [3].

Typical molecular weights of HA that have been studied in several biomaterials applications are: (C_14_H_21_NO_11_)_n_, 5 kDa, 60 kDa, 800 kDa and 3000 kDa [4]. However, HA polymers having >1.8 MDa molecular weight have been made commercially available. HA possesses exceptional physicochemical properties such as biocompatibility and biodegradability, non-inflammatory, non-toxicity and non-immunogenic behavior [3,5]. HA is a common material in many medical applications, such as visco-supplementation, eye surgery and drug delivery [6,7]. HA and HA derivatives have been utilized in in vitro and in vivo models for their ability to optimize the delivery of drugs belonging to different classes. Some examples are antibiotics such as gentamicin, antiglaucoma drugs like pilocarpine and betaxolol, the vasodilator serotonin (5-hydroxytryptamine), the cytokine interferon and the enzyme thrombin, to name but a few [8,9,10].

A recent review by Fallacara [11] described and documented thoroughly the history of HA and its physicochemical, structural and hydrodynamic properties, occurrence, biosynthesis, degradation mechanisms and receptors. They also presented advances in the industrial production of HA and its chemical derivatization. They mentioned applications like the cosmetics potential of HA [11]. Similarly, Knopf-Marques et al. [12] compiled an extensive review on the use of HA and HA derivatives in the implantable biomaterial microenvironment. They focused on materials such as hydrogels and special coatings that can deliver cytokines to decrease adverse immune reactions and promote tissue healing [12].

**Scheme 1:**
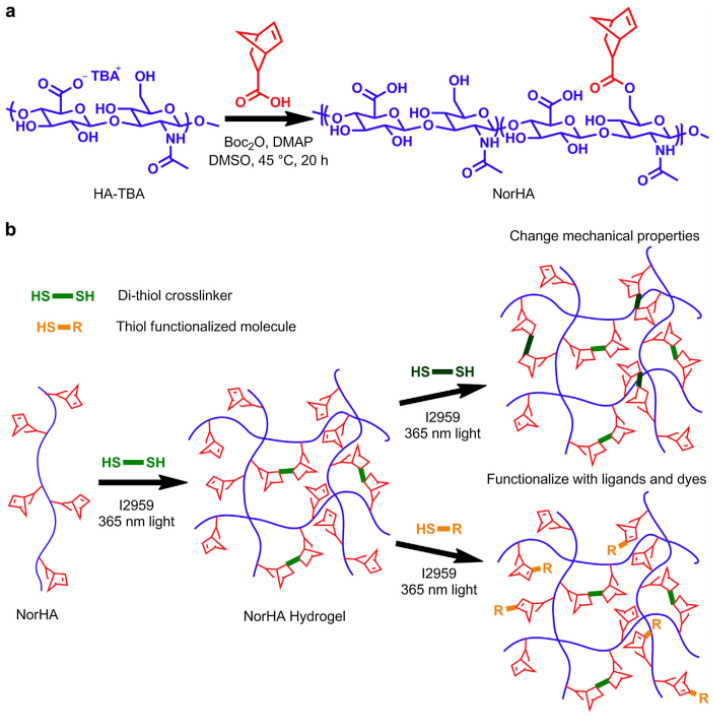
(**a**) Norbornene-modified (NorHA) HA hydrogel from the salt form HA-TBA through the coupling of norbornene carboxylic acid to pendant alcohols on HA. The reaction is realized by a di-*tert*-butyl decarbonate-activated process (**b**) Synthesis of gels via light-initiated thiol-ene reaction between a di-thiol and NorHA with subsequent chemical modification with mono- and/or di-thiols. From [21].

HA is also very important as an effective lubricant in the biomechanics of the moving joints of animals and humans alike [13,14]. HA can display anti-arthritic effects through multiple mechanisms involving receptors, enzymes and other metabolic pathways. HA can also be reacted or cross-linked in order to tune its chemical properties and resistance to aqueous dissolution [15]. Šmejkalová et al. [16] indicated that modified HA or HA derivatives possess certain advantages over pristine HA such as higher resistance against enzymatic degradation and delayed aqueous dissolution by attaching hydrophobic functional groups. Amphiphilic HA derivatives can be ideal platforms for hydrophobic drug encapsulation and delivery as well [17,18,19]. By esterification and modification of the degree of substitution and the length of attached carbon chains, HAs can form supramolecular assemblies that can be used for specific pharmaceutical applications. Modified HAs appear to have a great potential as novel drug carriers in the form of conjugates. The absence of positive charges on certain HA derivatives could alleviate the problems associated with severe cytotoxicity and aggregation with serum proteins in the body [16]. Several unique HA derivatives have been synthesized to date. For instance, HA can be functionalized with norbornene groups [20] and reacted with dithiols to formulate biocompatible hydrogels with tunable mechanical properties (see Scheme 1). Prior to that, HA needs to be transformed into a salt form known as tetrabutylammonium salt HA (HA-TBA).

HA can be cross-linked by other methods as well, such as water-soluble carbodiimide [21] to produce insoluble films when brought into contact with water. Studies indicated that intermolecular formation of ester bonds between the hydroxyl and carboxyl groups belonging to different polysaccharide molecules caused crosslinking. Carbodiimide crosslinking of HA in the presence of l-lysine methyl esters was also shown to prolong the in vivo degradation of HA [21].

Since the focus of this review article is on the release of pharmaceutical agents such as antiseptics and natural antioxidants or medical drugs from HA and the release of HA itself from other biomedical materials, it is important to briefly review recent advances in chemical reactions that are used to modify HA such as copolymerization, crosslinking, grafting, hydrogel formation, etc. In fact, very recently, Trombino et al. [15] comprehensively reviewed such reactions of HA, predominantly the hydrogel formation. For this reason, the review will just touch briefly on these reaction systems and focus more on the published studies on controlled release dynamics involving HA or its derivatives. We note that HA is also extensively used to improve the biocompatibility properties or bio-lubrication properties of other polymers [22,23,24,25]. For instance, Palumbo et al. [26] made graft copolymers between HA as a hydrophilic backbone and polylactic acid (PLA) as an aliphatic polyester in order for HA to be able to hydrophobically associate in an aqueous medium.

**Scheme 2:**
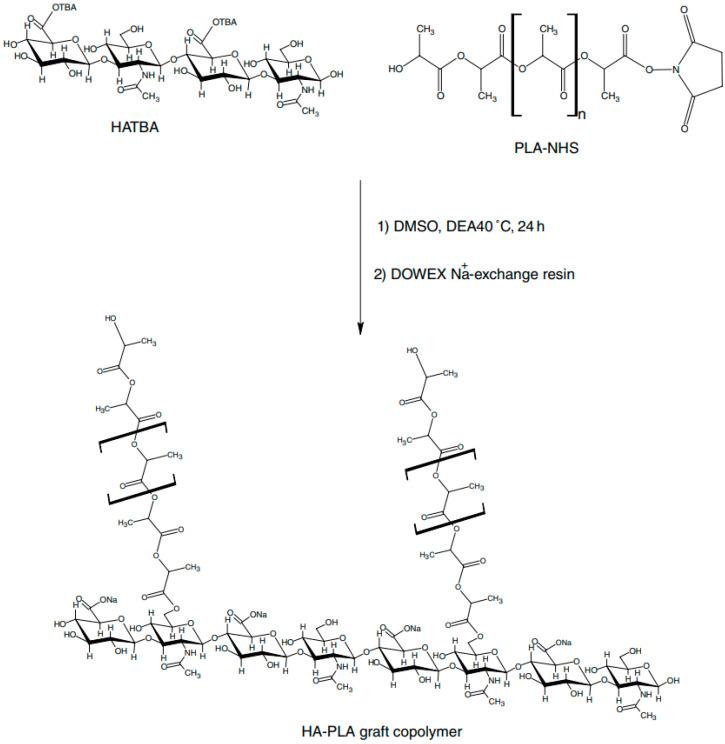
Schematic representation of the reaction between HA–TBA and PLA–NHS to give HA–PLA graft copolymer [26].

To achieve this, low molecular weight HA was made soluble in an organic solvent by transforming it to tetrabutylammonium (TBA) salt (HA–TBA). With this derivative, the reaction was performed in dimethylsulfoxide by adding N-hydroxysuccinimide modified PLA. The reaction is schematically depicted in Scheme 2.

**Figure 2:**
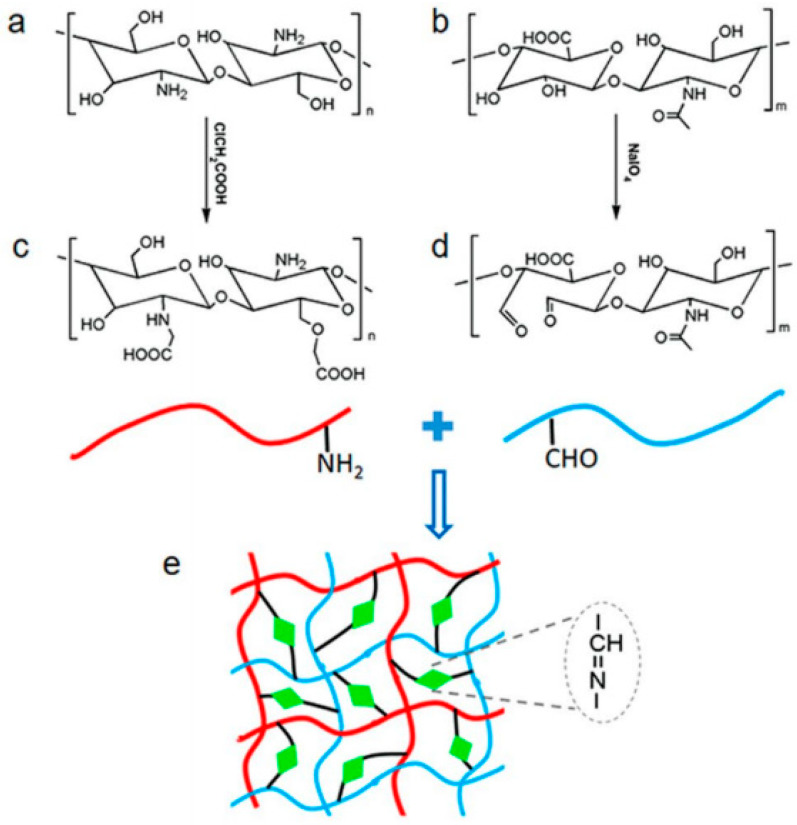
Schematic illustration of preparation of CS/HA hydrogel via Schiff’s base reaction. Chemical structures of chitosan (CS) (**a**), carboxymethyl chitosan (NOCC) (**c**), hyaluronic acid (HA) (**b**), and aldehyde hyaluronic acid (A-HA) (**d**). The employed fibrin gel or CS/HA hydrogel (green arrow: fibrin gel or CS/HA hydrogel (**e**). From [29].

In fact, a comprehensive review by Palumbo et al. [27] presented graft polymers of HA and various other polymers, with synthesis, properties, and applications. The difficulty in chemical modification or synthesis of HA derivatives is due to intermolecular entanglement, and complications associated with the control of viscoelasticity and molecular weight properties. A general method for inducing chemical modification to HA involves using a carboxylic acid (COOH) and an alcohol (OH) functional groups that are present in a repeating unit of a polymer or a physical method by using a carboxylic acid anion charge [28]. Recently, Deng et al. [29] reacted chitosan (CS) and HA by modifying both natural polymers chemically to form hydrogels. N, O-carboxymethyl chitosan (NOCC) was synthesized by carboxymethylation of CS. The carboxymethyl groups were introduced into the N-terminal and O-terminal of CS. The measured substitution degree of NOCC was 95% with good water solubility in PBS (pH 7.4). Aldehyde hyaluronic acid (A-HA) was made by oxidation reaction of HA using NaIO_4_. The hydroxyl groups of HA were oxidized to dialdehydes by opening the sugar rings to form dialdehyde derivatives with an oxidation level of 48.9%. The mechanism of gelation was obtained by Schiff base reaction between amino group of NOCC and aldehyde groups of A-HA as shown in Figure 2 [29]. These gels were successfully employed in abdominal tissue regeneration.

Tomihata and Ikada [30] reported chemical crosslinking of HA with poly(ethylene glycol) diglycidyl ether (a diepoxy compound) to limit the water uptake of HA and slow down its degradation upon contact with water. The minimal water uptake of the cross-linked HA films obtained was 60 wt.% when swollen with buffered saline at 37 °C. This particular film had in vivo 30% weight loss after 7 days of subcutaneous implantation in rats with reduced inflammation. As can be seen, the ability to formulate HA derivatives with diverse chemical functional groups greatly facilitates HA crosslinking to form novel hydrogels and the conjugation of biologically active factors such as drugs, growth factors, cytokines, etc., into HA can enable sustained release of drugs [31]. For instance, in a highly cited work, Bulpitt and Aeschlimann [31] introduced functional groups to HA by formation of an active ester at the carboxylate of the glucuronic acid moiety and successive substitution with a side chain containing a nucleophilic group on one end and a (protected) functional group on the other. As a result, HA derivatives with amino or aldehyde functionality were obtained along with hydrogels having bi-functional cross linkers. Mixtures of HA derivatives could also be made by carrying different functionalities using active ester- or aldehyde-mediated reactions [31].

An important issue that needs to be addressed is that any type of new HA derivative needs to be checked and verified against clinical safety regulations as well as tolerability in terms of tissue reactions. Even though high molecular weight HA injection dosages for treating bone damages, for instance, are known to be safe in terms of inflammation response or toxicity [32], in certain cosmetic treatments, injected HA systems did cause severe granulomatous allergic tissue reactions [33]. Even minute amounts of certain protein contaminants in HA-based release systems can cause granulomatous tissue reactions [34]. Although cutaneous granulomatous reaction to injectable HA gels is known to occur after cosmetic soft tissue augmentation operations, such cutaneous lesions could spontaneously disappear without leaving any scars [35]. This demonstrates that HA hydrogels are quite safe even if some skin reaction complications can develop but no long term severe clinical effects and scars have been reported to date.

Although chemical modifications and reactions of HA and its derivatives are beyond the scope of this review, it is acknowledged that design and synthesis of innovative HA derivatives for biomedical applications are exceedingly important for drug efficacy and targeting. The numerous chemical modification methods summarized above demonstrate that there exists a broad spectrum of options for the synthesis of new HA derivatives with various physicochemical properties. Readers can refer to several excellent reviews on this subject such as Schanté et al. [5].

Finally, before looking at several sustained release material systems based on HA and its derivatives, it is acknowledged that design of sustained release systems requires a thorough understanding of mathematical modeling of drug release from polymeric matrices and interactions between drugs and polymers [36]. In general, drug release from hydrogels are modeled to account for both diffusion (Fickian diffusion) and desorption (based on Langmuir kinetics) mechanisms that control the overall release rate. Generally, infinite sink, i.e., absorbing boundary conditions are used [37]. The comprehensive review by Lin and Metters [38] can be consulted for mathematical modeling of drug release from various hydrogels including polysaccharides and HA. As an example, Kaya et al. [39] fabricated electroconductive polymeric films made up of a HA composite containing gelatin, poly(ethylene oxide) and reduced graphene oxide as a release matrix. Release kinetics of irbesartan, a drug for cardiovascular disease treatment, was both modeled and experimentally measured for model verification. They used different dynamic differential mathematical models such as 1st, 2nd, 3rd degree and Higuchi model. All models estimated drug release kinetics reasonably well and the authors also proposed an alternative model derived from Higuchi model [39].

The aim of this review, however, is to highlight the many possibilities offered by HA and HA derivatives with mono- and polyfunctional chemical moieties that can lead to biomedical materials with sustained drug release capabilities such as biochemical probes, biopolymers with on-demand responsive drug release (on-off switching release by local heat, light or mechanical stress application), tethered drugs for controlled release, and cross-linked macro/nano hydrogels as biocompatible scaffoldings for tissue engineering [40]. We will start with reviewing advances made in the release of HA from other biomaterials since HA has unique intrinsic therapeutic properties and then we will review works on sustained release of proteins and related biomolecules, antiseptics and antibiotics and conclude by presenting the state-of-the-art in sustained release of cancer and tumor-targeting drugs from HA and its derivatives.

## 2. Controlled Release of HA from Other Biomaterials

As was demonstrated in the previous section, HA is a very important and versatile biomedical polymeric matrix. However, it is also a very important biological material that can function as “therapeutic agent” because HA can trigger various cell responses [32]. High molecular weight HAs (>5 MDa) demonstrate anti-angiogenic and immunosuppressive effects. Medium-size HAs (20 kDa–1 MDa) are associated with embryogenesis, wound healing and regeneration. Small-size HAs (6 kDa–20 kDa) are associated with pro-inflammatory, angiogenesis and gene expression processes. Small strands of HA function as anti-apoptotic agents and inducers of the heat shock proteins [32]. Hence, this chapter focuses on HA delivery and release studies from other biomaterials.

**Figure 3:**
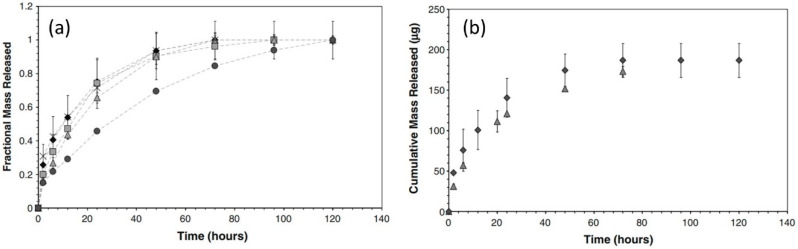
(**a**) Fractional release of HA from imprinted Nelfilcon hydrogels with different proportions of functional monomers. Dynamic release profiles were normalized with the total amount of HA released by hydrogels prepared from different proportions of functional monomers [AM:NVP:DEAEM], all at 0.125%-by-mass of prepolymer (monomers mixture): [1:1:0] (gray squares), [1:1:2] (black diamonds) and [0:0:1] (crosses). For comparison, the fractional release profiles of hydrogels with 0.25% [1:1:2] (circles) and hydrogels with no functional monomers (gray triangles) are also plotted. (**b**) Cumulative Release of HA from Nelfilcon hydrogels before and after heat-sterilization. When hydrogels containing HA with Nelfilcon were heated to 121 °C, the dynamic release profile of HA for such lenses (gray triangles) was similar to the release profile from hydrogels that did not undergo heat treatment (gray diamonds). From [41].

Probably, one of the most important need to release HA is related to supplying certain tissues of soft bone joints with proper biological lubrication and tribological function [41]. For instance, dry eye diseases could be addressed by replenishing HA in the eye. This could be achieved by controlled release of high molecular weight HAs from contact lenses [26]. Ali et al. [41] prepared hydrogel films and contact lenses made by nelfilcon A, acrylamide (AM), N-vinyl pyrrolidone (NVP), and 2-(diethylamino) ethyl methacrylate (DEAEM) and biomimetically imprinted them in the presence of HA to allow for controlled release of HA over 24 h-period. Their lenses were designed for the therapeutic delivery of HA to the eye to improve the wettability of lenses and to treat symptoms of dry eye. They showed that they could control the release rates of HA with two distinct methods. In the first, they could vary the cumulative mass released by varying the total amount of functional monomers added to the contact lens hydrogel, and as a second, they manipulated the diffusion coefficient by varying the diversity of incorporated monomers. Some representative results are given in Figure 3.

The summary of the results presented in Figure 3 can be given as follows: HA can be released and delivered from any commercial daily disposable lens at a therapeutic rate of about 6 μg/h for 24 h. The authors claimed that their work is among the first that demonstrates imprinting large molecular weight HA within a hydrogel contact lens and that they could tune the reptation (thermal motion of very long linear, entangled macromolecules in polymer melts or concentrated polymer solutions) HA to adapt to the lens hydrogel structure.

Similarly, Maulvi et al. [42] presented two methods to load HA in hydrogel contact lenses: soaking method and direct entrapment. HA-based hydrogels made by a soaking method released HA up to 48 h with acceptable physical and optical properties. The ones prepared with direct entrapment method showed significant longer/sustained release profiles compared to the soaking method. They also made in vivo pharmacokinetics studies with direct entrapment lenses in rabbit tear fluid that showed intense increase in HA mean residence times and area under the curve with lenses in comparison to eye drops treatment [42]. Fagnola et al. [43] designed two experimental spectroscopic procedures to determine the content of HA release from hydrophilic contact lens matrices. HA was released into aqueous solutions and the solutions contained cationic dyes to form complexes with HA with sensitivity down to concentrations of either 0.25 or 2.5 μg/mL. They claimed that their process can be utilized also for commercial contact lenses to distinguish among contact lens properties either HA loaded or unloaded. They indicated certain applicability practices to determine the content of HA in the ones that contain HA, to evaluate the release of HA by the lens in solution, and to understand the role of HA in preventing adsorption and successive release of other biological compounds from the contact lenses.

**Figure 4:**
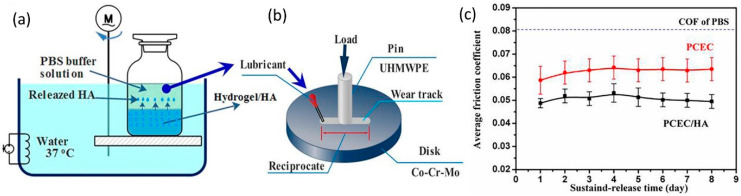
(**a**) HA release test from the hydrogel and (**b**) removing the liquid to measure its properties as a lubricant using a tribometer (**c**) Average friction coefficients of the sustained-release HA solutions for 8 days. COF—Coefficient of friction, UHMWPE—Ultrahigh molecular weight polyethylene. From [45].

Joints also need to be constantly lubricated for health articular cartilage function. For treatment of intra-articular regions and administration of medical compounds, sustained-release formulations are desirable since it is difficult for the joints to endure the effect of conventional injections due to drug leakage from the joint cavity [44]. Guo et al. [45] applied a temperature-sensitive poly(ɛ-caprolactone)–poly(ethylene glycol)–poly(ɛ-caprolactone) (PCEC) hydrogel containing HA to achieve a long-term lubrication by sustained release of HA. Their experimental results revealed that the friction coefficient of the released solution from PCEC hydrogel was about 38% lower than that of phosphate buffer saline, furthermore the ability of shear resistance and creep recovery of HA releasing hydrogel was found to be better than that of PCEC hydrogel alone, as shown in Figure 4. They claimed that such a material releasing HA into joint fluids could achieve long-time lubrication effect for artificial joints.

As mentioned earlier HA is also a key cosmetic therapeutic agent for skin but also is naturally found in our skins and helps heals skin wounds. Kasetvatin et al. [46] used a synergistic approach to enhance transepidermal delivery of HA using elastic liposomes (ELs) and low frequency ultrasound (LFU). HA-loaded ELs were made by changing the cholesterol contents by a reverse phase evaporation technique. In vitro permeation studies were conducted using porcine ear epidermis. In addition, skin barrier disruption was assessed by transepidermal water loss and histology studies. The LFU exposure time was associated with both drug penetration and skin perturbation. As the exposure time was increased, the skin perturbation increased. They found that 1 min exposure optimally enhanced HA permeation without damaging the epidermis.

**Figure 5:**
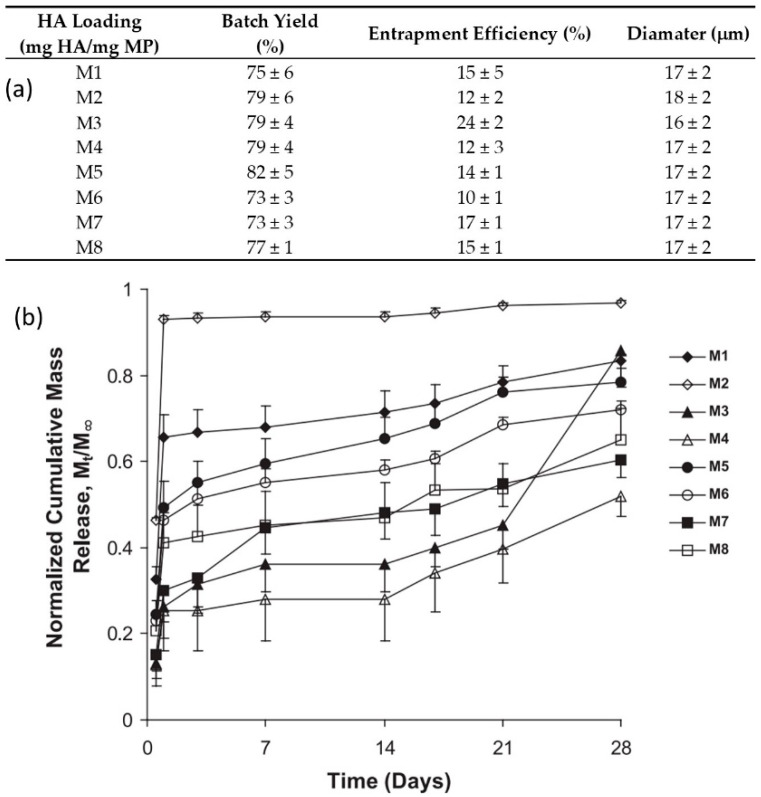
(**a**) Summary of microparticle (MP) properties. In their notation, HY stands for hyaluronic acid (HA). (**b**) Cumulative release of HA (or HY in their notation) from PLGA/PEG microparticles in PBS at 37.8 °C. The release is expressed as normalized mass release. The results are presented as means with ± standard deviation for five measurements. M1, M3, M5 and M7 micro-particles were produced by the higher molecular weight HA (see above text). From [49].

As can be seen, an important application of HA is its use in transdermal delivery. However, most studies on transdermal delivery of HA from other vehicles such as phospholipid-conjugated SPACE peptides [47], natural polymers or gels do not study controlled release properties and kinetics but rather focus on penetration or diffusion of high molecular weight HA into skin [48], which is a difficult barrier for diffusion of macrobiomolecules. There are very few works on the controlled release of high molecular weight HA from micro or nanostructured biopolymer systems [49]. Among them a notable work was conducted by Hedberg et al. [49] in which biodegradable microparticles of blends of poly(d,l-lactic-co-glycolic acid) (PLGA) and poly(ethylene glycol) (PEG) were prepared as a potential carrier for the controlled release of HAs of variable molecular weights. They used two HA oligomers of molecular weights Mw = 15 kDa and Mw = 49.5 kDa. They showed that modifications in PLGA microparticle formulations resulted in alterations in the release kinetics of HAs of different molecular weights. Their formulations, regardless of their differences, indicated three regions of release: a burst release that lasted for one day, a linear release trend or region between the first day and day 14, and a second linear release zone from day 14 until day 28 where the measurements were terminated. PEG concentration changes within the microparticle showed no effect on the cumulative mass released during the initial burst release but affected the release rates during both the first and second regions of linear release. The initial HA concentrations in microparticles and the PLGA molecular weight, as well as the molecular weight of HA modulated all three-region release trends observed in their study (see Figure 5) [49]. In Figure 5b, the cumulative mass release of HA for each time-point (M_t_) was calculated and normalized by the total amount of HA loaded in within the microparticles (M_∞_).

Osorno et al. [50] produced poly(d,l-lactic-co-glycolic acid)-b-poly(ethylene glycol) triblock copolymers (PLGA-PEG) matrices to encapsulate HA and studied its controlled release. They focused on copolymer gel formation and mechanical properties on release kinetics of HA. They have modulated several processing and materials properties such as hydrophilic/hydrophobic ratio (i.e., PLGA/PEG ratio), lactic acid/glycolic acid ratio, PEG molecular weight, copolymer molecular weight, and polydispersity index to optimize controlled release of HA.

Because of this optimization process, they designed an injectable, thermoresponsive hyaluronic acid (HA) delivery platform for ocular applications. They have also claimed that an extended and controlled release of HA, beyond 2 weeks could be built from injectable hydrogels modified with a noncovalent interacting agent, poly(l-lysine) or PLL. Smaller PLL chains slowed down the HA release kinetics, while larger PLL chains produced a release profile similar to the unmodified hydrogels [50].

HA mostly encountered in vertebrate soft tissues (e.g., joints, synovial fluid, skin, vitreous humour of the eye, umbilical cords, roster combs), in algae, in molluscs, also in cultured eukaryotic cell lines, and certain prokaryotes. HA is the major constituent in the vitreous of human eye (0.1 mg/mL wet weight), and in the synovial joint fluid (3–4 mg/mL wet weight). There exist about 7–8 g of HA in our bodies and about 50% of this is found in the skin distributed between the dermis and the epidermis (0.5 mg/g wet tissue) [51]. Therefore, HA based controlled released materials become very attractive since the likelihood of its in vivo rejection is extremely low. In the following sections, we will review HA based controlled release matrices that have been designed to deliver certain pharmaceutics such as antiseptics, antibiotics, antioxidants, in addition to lipids, proteins, peptides, DNA, hormones, and electrolytes etc. in a sustained manner both in vitro and in vivo.

## 3. Controlled Release of Proteins from HA

Effective use of proteins as drugs is by no means new. Common examples that have been routinely employed for decades are insulin, gamma-globulin and protein-containing vaccines. Nonetheless, new pharmaceutical technologies including nanoscale drug delivery have triggered a dramatic increase in the development and use of proteins as novel drugs. The technology is such that virtually any desired protein in sufficient quantities could be synthesized and purified for therapeutic use today. It is however more challenging to keep proteins stable and encapsulate them in proper matrices without loss of stability. Proteins are intrinsically less stable than many other organic pharmaceutical low molecular weight active principles. As such, it remains a continuing challenge to formulate proteins that can be handled without damage throughout their entire lives. Hence, matrices that can keep proteins active and structurally stable until it is delivered to the relevant in vivo site of action with maximum efficiency are highly sought [52].

Pure or modified HA can effectively encapsulate proteins and allow their controlled release for various potential medical treatments [53]. For instance, hydrophilic HA particles can encapsulate growth factors, regulating their release and potentiating their biological functions. Xu et al. [54] designed heparin (HP)-decorated, HA-based hydrogel particles (HGPs) using an inverse emulsion polymerization technique with divinyl sulfone crosslinker. Their microscopic particles were spherical in shape and contained nanosized pores suitable for growth factor encapsulation. The covalently attached HP retained its ability to bind bone morphogenetic protein-2 (BMP-2) specifically, and BMP-2 release kinetics were adjusted by changing the particle composition. They also showed that compared to pure HA particles, the hybrid HA/HP hydrogel particles had a higher BMP-2 loading capacity. BMP-2 was released from HA HGPs with a significant initial burst but a near zero order release kinetics was observed from HA/HP hybrid particles with an optimized heparin content of 0.55 μg per mg HGPs. They argued that the simplicity of their synthesis protocols, and the materials chosen can render such hydrogel particles an attractive candidate for the sustained release of BMP-2, targeting cartilage repair and regeneration [51]. Similarly, Kim and Park [55] developed temperature-sensitive HA hydrogels using photopolymerization of vinyl-modified HA in combination with acrylate group end-capped poly(ethylene glycol)–poly(propylene glycol)–poly(ethylene glycol) tri-block copolymer, known as Poloxamer 407. These hydrogels were shown to gradually collapse with increasing temperature within the range 5–40 °C, indicating that Poloxamer 407 formed self-associating micelles in the hydrogel structure. Incorporation of recombinant human growth hormone in these HA hydrogels produced sustained protein release followed by a mass erosion or biodegradation. The authors did not specifically indicate a specific target treatment or administration route but in general, temperature responsive hydrogels are developed for increased circulating time in the blood stream after intravenous administration. Such hydrogels can conform to different self-assembled morphologies in the blood stream triggered by mild temperature changes.

**Figure 6:**
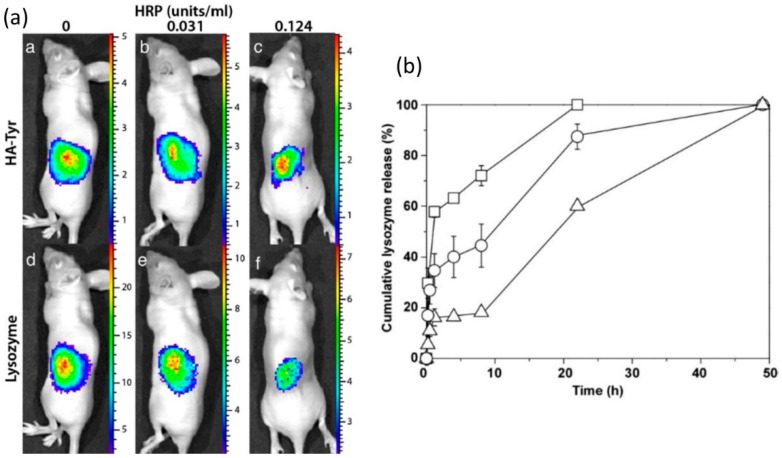
(**a**) In vivo formation of HA–Tyr hydrogels containing lysozyme. H_2_O_2_ concentration was fixed to 728 μM while the concentration of HRP increased from 0 (a,d) to 0.031 (b,e) to 0.124 (c,f) U/mL. The upper row (a–c) shows the locations of HA–Tyr and the lower row (d–f) shows the location of lysozyme. Color bar has the unit of 10^9^ photons/s/cm^2^/steradian. (**b**) Cumulative release of lysozyme from HA–Tyr hydrogels in the presence of 2.5 U/mL of hyaluronidase. The hydrogels were formed with 0.124 U/mL of HRP and (□) 473, (○) 582 and (△) 728 μM of H_2_O_2_ (*n* = 2, mean ± S.D.). From [56].

HAs are also ideal materials for injectable hydrogels. A novel injectable hydrogel was made by Lee et al. [56]. The hydrogels were made by the oxidative coupling of tyramines (HA-Tyr) that were catalyzed by hydrogenperoxide (H_2_O_2_) and horseradish peroxidase (HRP). They argued that the rapid gelation by optimizing the concentration of HRP could effectively encapsulate the proteins and prevent undesired leakage of proteins into the surrounding tissues after injection. Hydrogels with different mechanical properties were made by changing the concentration of H_2_O_2_ while maintaining rapid gelation. Stiff hydrogels released proteins slower compared to softer ones. In phosphate buffer saline, α-amylase (negatively charged) displayed sustained release characteristics. Conversely, the release of lysozyme (positively charged) stopped after 4 h due to electrostatic interactions with HA. They also showed that in the presence of hyaluronidase, lysozymes in the subcutaneous environment (see Figure 6a) were released continuously and completely from the hydrogels as the gel network rapidly degraded (see Figure 6b). The activities of the released proteins were retained rending these hydrogels ideal for the delivery of therapeutic proteins.

Lee et al. [57] fabricated HA nanogels (200 nm in diameter) by an inverse emulsion/crosslinking method with ultrasonic processing. Crosslinking was done by forming disulfide bonds between the HA chains. Green fluorescence protein (GFP) small interfering RNA (siRNA) was encapsulated in the HA nanogels with no structural protein damage. The controlled release of SiRNA from HA gels was modulated by glutathione (GSH) concentrations in the buffer solution. The authors also showed that HA nanogels were transported into the cells by CD44 receptor mediated endocytosis [57]. Cellular uptake and gene silencing effects of siRNA/HA nanogels were also studied by the researchers [57]. Famili and Rjagopal [58] fabricated chemically cross-linked hydrogels for protein delivery using a catalyst-free inverse-demand Diels–Alder reaction between tetrazine and norbornene chemical groups. The cross-linking chemistry was shown to be chemically inert to proteins. Specifically, they used tetrazine-modified hyaluronic acid and norbornene-modified polyethylene glycol as hydrogel precursors for in situ encapsulation of a model protein, 1-phosphatidylinositol 3-phosphate 5-kinase (Fab1). They could tune gelation kinetics and subsequent gel stiffness using temperature but independent from Fab1 concentration. In vitro release testing indicated that Fab1 was entirely released from the hydrogel over a period of several weeks. They also showed that released Fab1 protein did not undergo any physical or chemical modifications and retained its antigen binding capacity.

**Figure 7:**
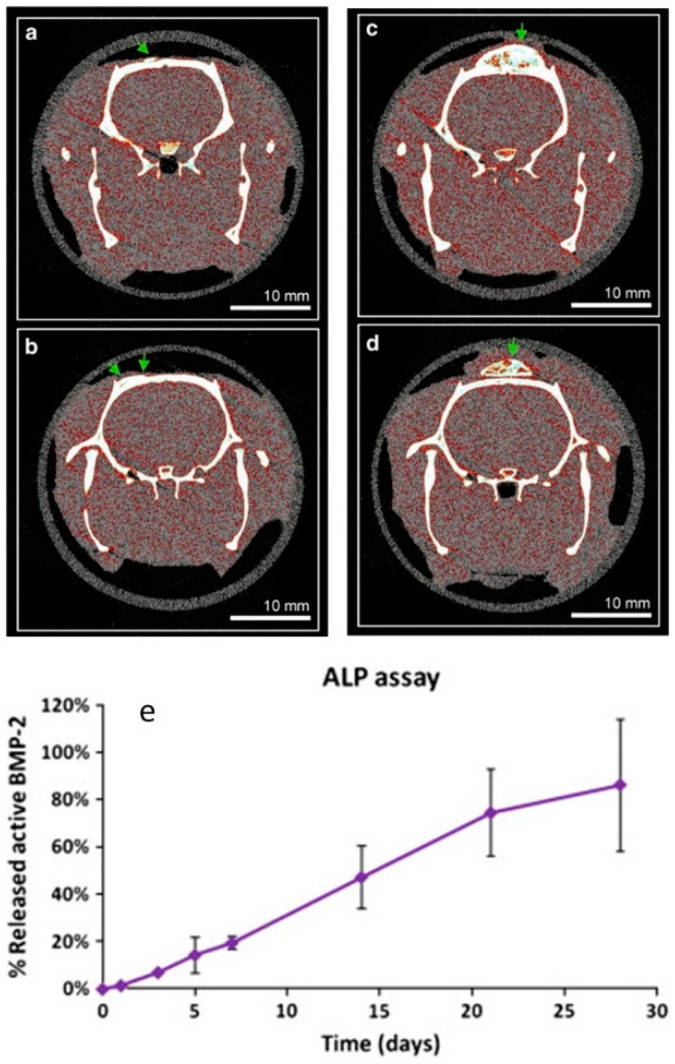
Representative quantitative computerized tomography (pQCT) coronal section images of injected hydrogel (200 μL) after 8 weeks. (**a**) subperiosteal injection without rhBMP-2; (**b**) subperiosteal injection with 5 μg/mL rhBMP-2; (**c**) subperiosteal injection with 150 μg/mL rhBMP-2; and (**d**) subcutaneous injection with 150 μg/mL rhBMP-2. Arrows indicate the location of a newly formed bone. (**e**) Controlled release profile of active rhBMP-2 from HA gels up to 28 days of incubation in vitro. From [60].

Leach and Schmidt [59] incorporated a model protein, bovine serum albumin (BSA), into photopolymerizable glycidyl methacrylate-hyaluronic acid (GMHA) and GMHA–PEG hydrogels. They studied the diffusional properties of BSA within the hydrogels as well as the effect of the photo-crosslinking kinetics on protein aggregation. To achieve sustained protein release, they fabricated hydrogel–microsphere composite release systems by dispersing BSA-containing poly(lactic-co-glycolic acid) or PLGA microspheres within the hydrogel solution prior to crosslinking. Their results indicated that GMHA-based hydrogels and hydrogel–microsphere composites could be suitable systems for delivering stable proteins in soft tissue engineering applications. Martínez-Sanz et al. [60] worked on the synthesis of hydrazone cross-linked HA hydrogels that were made under mild reaction conditions. They also demonstrated in vivo application as recombinant human protein (rhBMP-2) carrier for bone formation in the rat calvarial model (see Figure 7a).

The model was chosen specifically to address autologous cranial bone grafts that are widely used for the reconstruction of several bone defects such as atrophy of the human jaws in dentistry. They demonstrated that calvarias bone augmentation could be achieved by using injectable HA hydrogel with different amounts of rhBMP-2 below the subperiosteal space. The rhBMP-2 concentrations in the experiments were increased from 5 μg/mL (Figure 7b) to 150 μg/mL (Figure 7c) and also 150 μg/mL for the subcutaneous injection in Figure 7d. To explain the mechanism of in vivo bone formation they performed in vitro experiments that demonstrated controlled release of active rhBMP-2 through model scaffolds within 28 day-period (see Figure 7e). Their hydrazine-based HA hydrogels were proven to be biocompatible by performing in vitro cytotoxicity assay.

**Figure 8:**
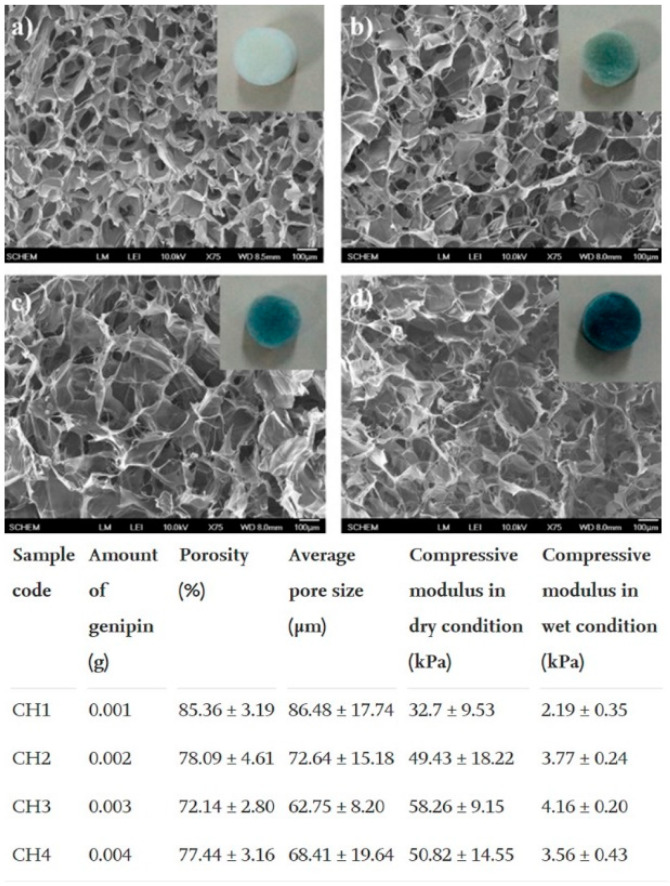
SEM images of only chitosan (**a**), CH1 (**b**), CH3 (**c**), CH4 (**d**) scaffolds. See table below for material details and mechanical properties. The scaffold is structured by chitosan, whereas the extracellular matrix (ECM) part is obtained by HA. Images of each type of scaffold (cylindrical shape with 1 cm in diameter) have been shown inset. From [62].

Hahn et al. [61] developed an injectable controlled release formulation of erythropoietin (EPO) encapsulated in selectively cross-linked HA micro-hydrogels. EPO is a bioactive protein that regulates red cell production by promoting erythroid differentiation and initiating hemoglobin synthesis. This protein also has neuroprotective activity against a variety of potential brain injuries and antiapoptotic functions in several tissue types. The authors used adipic acid dihydrazide-grafted HA (HA-ADH) and then modified it into a thiolated HA hydrogel (HA-SH). EPO was incorporated in situ during HA-SH hydrogel synthesis using sodium tetrathionate that accelerated crosslinking reaction. In vitro release tests of EPO were conducted using liquid chromatography. The release tests were done using reversed-phase-high performance liquid chromatography (RP-HPLC). Using this technique even trace concentrations of proteins can be detected in aqueous media by passing the release medium through a high pressure tube containing specific adsorbents that can remove proteins from the flow system and in turn allows one to measure protein quantity in that particular liquid. They used reactive spray drying to form injectable HA-SH micro-particle hydrogels. Spray drying was made by keeping the gel temperature below 40 °C at an inlet temperature of 90 °C. Morphological structure of HA-SH microhydrogel was carried out with a microscope and its water content was measured with a thermogravimetric analyzer. They assessed the applicability of their materials for the controlled release of EPO in Sprague Dawley rats. Polyelectrolyte complex (PEC) is a polymeric solution in which polymers with opposite charges are combined. PECs are considered to be effective carriers for controlled release of drugs and proteins. Nath et al. [62] immobilized bone morphogenetic protein-2 (BMP-2) in a PEC system made up of chitosan and HA (see Figure 8).

Charge-to-charge stoichiometry of the PECs was estimated using turbidity measurements in solutions. Free amino groups in chitosan were cross-linked with different amounts of genipin. They studied degree of crosslinking, and its effects on swelling, degradation and cytocompatibility in vitro. Immobilization of three different amounts of BMP-2 in PEC scaffolds could cause sustained release of the growth factor for more than 30 days. Immobilization efficacies varied from 61% to 76% depending on the amount of BMP-2. Finally, they determined the effects in osteogenic differentiation of the PEC with BMP-2 to MC3T3-E1 cells by reverse transcriptase PCR.

Jiang et al. [63] synthesized ampholytic N-carboxyethyl chitosan (CEC), with various isoelectric points (IPs), by grafting acrylic acid on chitosan using Michael’s reaction. CEC had enhanced water solubility with fast enzymatic degradation compared to pure chitosan. The authors showed that the rate of degradation was proportional to the degree of substitution (DS). They further found using turbidimetric titration and fluorescence that CEC formed complexes HA and BSA within a certain pH range. They fabricated HA/CEC/BSA ternary complexes by colloid titration entrapping BSA protein. They measured the rate of BSA release from the complexes as a function of pH, ionic strength, DS of CEC, and the molecular weight of HA. They showed that the duration of BSA sustained release from the complexes could be maintained up to 20 days, formulating them with high molecular weight HA and CEC with low DS. Lim et al. [64] produced HA and polydopamine (PDA) complexes having hydrogen bonding interactions and tested these complexes as protein drug carriers. The complexes were formed with different molecular weight HAs (20 kDa and 200 kDa) and various molar ratios of dopamine and lysozyme, a model protein. Dopamine-conjugated HA (HADA)/PDA complexes (100–300 nm in diameter) were made by one-pot synthesis in which dopamine self-polymerization took place under oxidative, weakly basic conditions. Lysozyme was bound to HADA/PDA complexes via coacervation and hydrogen bonding.

The authors showed that during synthesis, protein structure and function remained unaltered. Namely, transition temperature of the HADA/PDA/lysozyme complex (1:10:0.05 ratio) was 72.45 °C, which is close to the melting point of the native lysozyme (72.46 °C). Protein encapsulation and efficacy of the formulations showed successful complexation as protein carriers, thus suggesting an effective combinatory protein delivery system.

**Figure 9:**
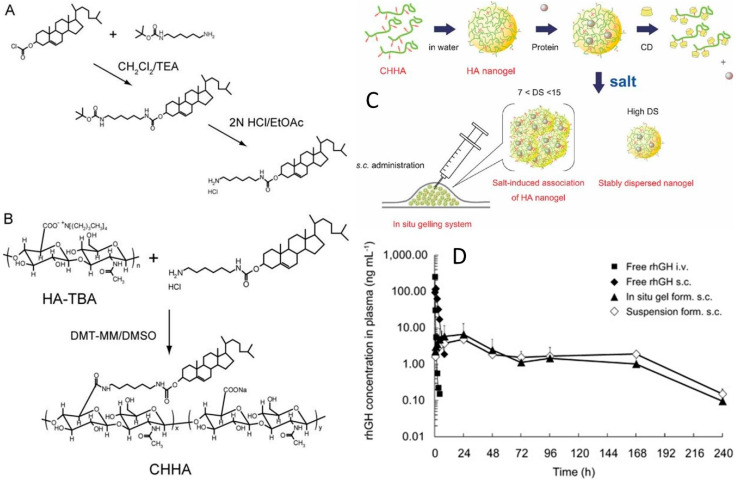
Synthesis of (**A**) cholesteryl-6-aminohexylcarbamate and (**B**) cholesteryl-group-bearing HA or CHHA, (**C**) schematic illustration of HA nanogels and solution properties, (**D**) pharmacokinetic study of HA nanogel formulation in rat. In situ gel formulation (solution state) and suspension formulation were injected subcutaneously into rats with a 25-gauge needle at a dose of 1.9 mg kg^−1^. Non-treated rhGH was also injected both intravenously at a dose of 0.1 mg kg^−1^ and subcutaneously at a dose of 0.5 mg kg^−1^. rhGH levels in plasma were determined using an ELISA. TEA: trimethylamine, HA-TBA: HA-TBA: Tetrabutylammonium salts of HA, DS: degree of substitution From [65].

Nakai et al. [65] fabricated HA based anionic nanogels from self-assembly of cholesteryl-group-bearing HAs (CHHA) designed for protein delivery, as shown in Figure 9. They transformed protein loaded HA nanogels into an injectable hydrogel formed by salt-induced association of the HA nanogel. CHHA was synthesized by condensing cholesteryl-6-aminohexylcarbamate with HA. Cholesteryl-6-aminohexylcarbamate was prepared as described in Scheme 1a. The synthesis route of CHHA is schematically shown in Figure 9B. CHHA formed a nanogel by self-assembly and salt-induced hydrogel was formed by the association of HA nanogel in the case of CHHA with DS of 7–15 as schematically shown in Figure 9C. The HA nanogels were shown to bind various types of proteins without denaturation, such as recombinant human growth hormone (rhGH), erythropoietin, exendin-4, and lysozyme. They also conducted a pharmacokinetic study in rats that showed that an in situ gel formulation, prepared by simply mixing human growth hormone, rhGH, and HA nanogels in phosphate buffer, maintained proper plasma rhGH levels within a narrow range over one week (see Figure 9D). Therefore, they claimed that the HA nanogels could offer a simple method to prepare therapeutic protein formulations that may be effective for sustained protein release applications.

Polyelectrolytes are commonly encountered in sustained drug release systems. They are simply polymers with ionizable groups on constituent monomers. Polyelectrolytes dissociate in polar solvents like water into polyions and oppositely charged moieties, low-molecular weight counterions. Polyelectrolyte complexes can be formed by electrostatic interaction of amino groups on chitosan polymeric chains with anionic groups such as carboxyl of other natural macromolecules or polymers like pectin, alginate, carrageenan, xanthan gum, carboxymethyl cellulose, chondroitin sulfate, dextran sulfate and HA. Chitosan can also form polyelectrolyte complexes with polyacrylic acid, polyphosphoric acid, poly(l-lactide). Hence, chitosan (CH) is considered to be one the best candidates for designing polyelectrolytes with other biopolymers. Polyelectrolyte complexes between CH and HA are commonly tested for skin, cartilage, and bone tissue engineering. However, their sustained release response in ionic media, like increased Ca^2+^ concentration near bone constructs is not completely resolved. To this effect, Krishna et al. [66] prepared freeze-dried scaffolds of CH-HA polyelectrolyte complexes, PECs, and characterized them by various spectroscopic and thermal techniques. FITC-conjugated BSA (albumin–fluorescein isothiocyanate conjugate), labelled as FA, was incorporated into the PECs to study the release properties in response to changes in Ca^2+^ concentrations. The authors also studied swelling of CH-HA in deionized water and aqueous Na^+^ and Ca^2+^ solutions. Swelling and FITC conjugated BSA (or FA) release were found to be high for the matrix in aqueous Ca^2+^ whereas it was unusually low in water and Na^+^. Sustain drug release rates were found to increase with concentrations of Ca^2+^ (0.02–1.0 M) indicating that CH-HA is a promising matrix for Ca^2+^ responsive delivery of proteins that can accelerate healing of bone defects that tend to release high amounts of Ca^2+^.

Purcell et al. [67] utilized the concept of synthetically sulfated HA’s ability to bind proteins with high affinity through electrostatic interactions. They explored the possibility of regulating protein release from HA hydrogels by modifying them with incorporation of sulfated HA. They developed sulfated and methacrylate-modified HA macromers and introduced them into HA hydrogels via free radical-initiated crosslinking. Note that a macromer is an assembly of pre-polymerized monomers that has been modified to act as a monomer by addition of one or more double bonds that can polymerize. They have much lower polymerization exotherms. Their work demonstrated that the sulfated HA macromers attached to a heparin-binding protein (stromal cell-derived factor-1 α, SDF-1α) with an affinity comparable to pure heparin and did not modify the gelation and network forming mechanisms when copolymerized into hydrogels at low concentrations. They further tested other materials systems in which the macromers were embedded into electrospun nanofiber hydrogels to introduce sulfate groups into macroporous scaffolds. They showed that the sulfated HA macromers considerably slowed encapsulated SDF-1α release over 12 days. They concluded that the macromers could be useful means to introduce heparin-binding features into cross-linked hydrogels to control protein interactions and release for desired applications. Zhang et al. [68] demonstrated an injectable cross-linked hydrogel system based on the reaction between HA derivatives and α,β-polyaspartylhydrazide (PAHy). The HA derivative having dialdehyde functionality (HAALD) was shown to react with PAHy under mild conditions forming hydrazine-conjugated hydrogels. No cross-linkers or catalysts were needed. The authors synthesized and characterized the HAALD-PAHy hydrogels in in PBSA solution (a biological buffer solution). The gels had porous morphology ideal for encapsulating proteins. Sustained and stable BSA release from the HAALD-PAHy hydrogels were observed during in vitro delivery experiments by the authors.

As can be seen from this section, there appears to be no specific HA-based system for a specific protein. For instance, BSA has been incorporated in different HA systems such as methacrylated HA or hydrazide modified cross-linked HAs to target general tissue engineering therapies. Most of the works reviewed herein focus on ensuring that the designed matrix should not denature the protein but also release it in a prolonged period. Table 1 below presents a summary of this section. It is evident from data of Table 1 that most of these reports achieved sustained protein release of a minimum of 2 days and in some cases up to a month without denaturing the protein. Shorter, few hours, release systems have also been reported but such studies are not in majority. Inspecting Table 1 further indicates that there are no correlations between the targeted therapy and the type of HA system developed. It appears that a significant portion of the works have focused on model protein release rather than determining a specific therapeutic target. In addition, almost all works have used modified HA material systems rather than pure HA. Till now, it is also not possible to conclude that nanoscale HA matrices demonstrate any sort of advantage over macroscopic, or microscopic scale matrices due to the arbitrary nature of the published reports.

## 4. Controlled Release of Antiseptics and Antibiotics from HA

Sustained release of antibiotics and antiseptics is of paramount importance. The reason is that to preserve antimicrobial activity, frequent administration of approved antibiotics and antiseptics with short half-life is necessary. Otherwise, minimum inhibitory concentration (MIC) levels can be exceeded during the treatment, which can cause antibiotic resistance. Maintaining a sustained release and adequate levels of antibiotics or antiseptics over MIC levels for a prolonged period will maximize the therapeutic effect of antibiotics while minimizing antibiotic resistance. In this sense, use of HA and its derivatives as matrices for sustained release of antiseptics and antibiotics is relatively new compared to other biopolymers such as chitosan, polyurethanes, polylactic acid derivatives and polyacrylates [69,70,71]. Hence, this section is devoted to the studies involving controlled release of pharmaceutical antiseptics and antibiotics from HA and HA-based biopolymer matrices. As will be shown, HA-based hydrogels, for instance, are excellent candidates for sustained delivery of antibiotics to even very delicate biological surfaces such as ocular tissues [72].

Romano et al. [73] reviewed the properties of HA and its composites as a local antimicrobial and antiadhesive barriers against biofilm formation on biomedical implants and surgical insertions. They demonstrated that antiadhesive properties of HA films or coatings are indeed ideal for bacterial biofilm formation that inherently provide an antibacterial barrier. Hsiao [74] et al. developed a sustained-release HA-based hydrogel targeting tendinopathy treatment. They formulated oxidized HA/adipic acid dihydrazide hydrogel encapsulating a drug. They studied the effect of HA on mitigating tendinopathy changes both in vitro (mechanically induced tendinopathy model) and in vivo (collagenase-induced tendinopathy model). They used a blend of epigallocatechin gallate (EGCG) and pigallocatechin gallate as the drug. Their results indicated the developed hydrogel and the powerful encapsulated antioxidants facilitated the healing process and mitigating oxidative stresses during tendinopathy treatment. Similarly, bone fracture fixation after trauma is connected to high infection rates. Antibiotic loaded biomaterials can target local zones with high concentrations without systemic side effects. To address this, Ter Boo et al. [75] reported thermo-responsive HA based hydrogels with gelation temperature control properties. The hydrogels were loaded with gentamicin and applied in an in vivo fracture model in the presence of fracture fixation hardware. The bacterial contamination was cleared in all of the inoculated rabbits in the presence of the antibiotic laden hydrogels. More specifically, hyaluronic acid-poly(*N*-isopropylacrylamide (HApN)) was prepared by direct amidation reaction between the tetrabutylammonium (TBA) salt of hyaluronic acid and amine-terminated poly(N-isopropylacrylamide) (pN). The degree of grafting, and gelation properties of the gel were studied including the gentamicin concentrations. They also tested release properties of the gentamicin-loaded HApN gels in vitro. The efficacy of the gel in preventing infection was tested in a rabbit model of osteosynthesis contaminated with *Staphylococcus aureus*.

**Figure 10:**
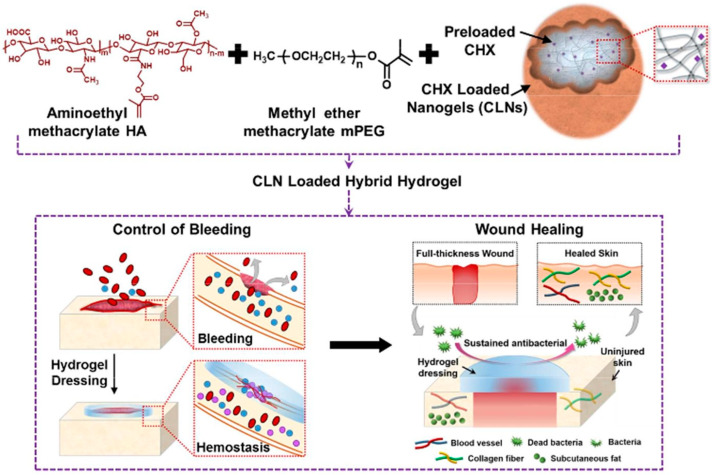
CLN-loaded hybrid hydrogel constructed from aminoethyl methacrylate HA, methyl ether methacrylate mPEG and CHX loaded nanogels (CLNs). The hydrogel enables controlled antibiotic release and accelerates wound healing. From [78].

Some antiseptic-loaded HA hydrogels are already commercially available and have found applications in clinical trials. Malizos et al. [76] used a commercial fast-resorbable hydrogel coating (Defensive Antibacterial Coating, DAC^®^; Novagenit Srl, Mezzolombardo, Italy) that is made up of covalently linked HA and poly-d,l-lactide and that can undergo complete hydrolytic degradation in vivo within 48–72 h while releasing a variety of different antibacterial agents at concentrations ranging from 2–10%. Valverde et al. [77] treated Ti-6Al-4V alloys with triclosan-loaded HA/chitosan multilayer films forming stable PEMs onto both smooth and laser micro-patterned alloy surfaces. Titanium surfaces became relatively hydrophilic. Both smooth and laser micro-textured surfaces were treated with the authors. Both surfaces, after modification, became reservoirs of bactericide for active delivery after implantation. Both multilayers could release about 25% of loaded triclosan within the first 10 h. Sustained release from these coatings inhibited bacterial adhesion and proliferation during the critical post-implantation period as verified by the authors against *Staphylococcus aureus* bacteria. Zhu et al. [78] constructed hybrid hydrogels with quick hemostasis and sustainable antibacterial action combining aminoethyl methacrylate hyaluronic acid (HA-AEMA) and methacrylated 2 methoxypolyethylene glycol (mPEG-MA) hybrid hydrogels and chlorhexidine diacetate (CHX) loaded nanogels (see Figure 10). Their aim was to control hemorrhage and anti-infection in wound management. CHX-laden nanogels (CLNs) were prepared by enzyme degradation of CHX loaded lysine-based hydrogels. The HA-AEMA and mPEG-MA hybrid hydrogel loaded with CLNs (Gel@CLN) featured a three-dimensional microporous structure with good swelling properties and low cytotoxicity. Gel@CLN hydrogels demonstrated sustained CHX release periods of up to 240 h with potent antibacterial affect lasting 10 days. The authors also evaluated hemostasis and wound healing properties in vivo using a mouse model. Their results indicated that hydrogels had fast hemostasis and accelerated wound healing potential.

**Figure 11:**
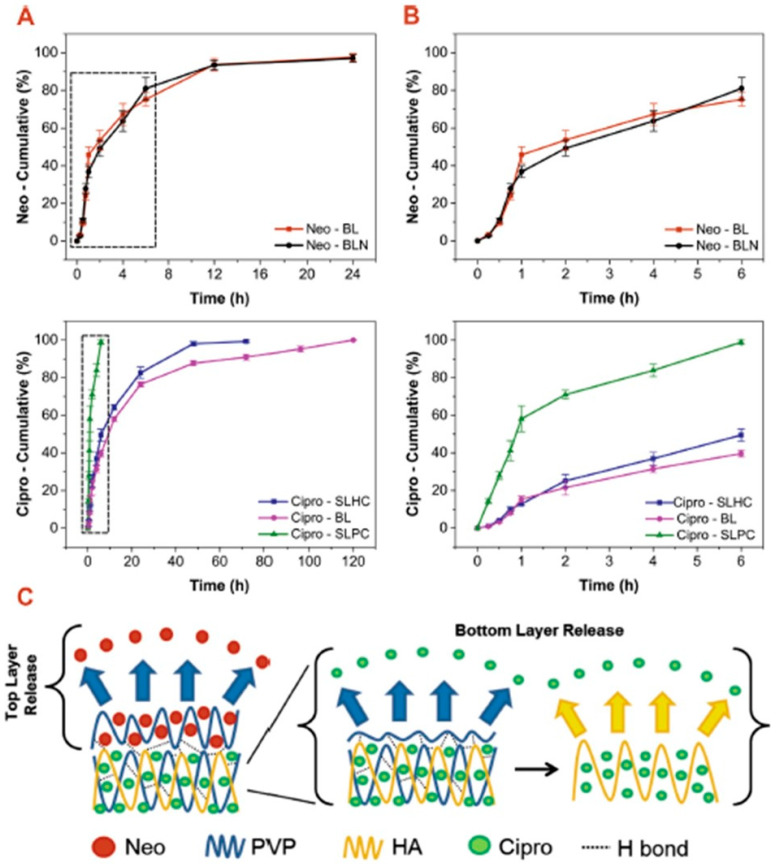
(**A**) Release profiles of eosin (top panels) and ciprofloxacin (bottom panels) over a period of 24 and 120 h, respectively. (**B**) Release results of eosin (top panels) and ciprofloxacin (bottom panels) for the first 6 h. (**C**) Schematic representation of the hypothetical drug release mechanism from the bilayer construct placed on a moist medium such as a wound. Potential establishment of hydrogen bonds within the top layer and at the interface between the top and bottom layers are shown as dashed lines. Neo: eosin based antiseptic. BL: full bilayer construct, BLN: the bilayer construct with no ciprofloxacin, SLHC: single layer with HA, SLPC: single layer without HA, and SLHP: single layer without ciprofloxacin. From [80].

Treatment of non-healing infected wounds could be achieved by antiseptic- and antibiotic-laden HA material systems. Curing non-healing wounds is based on early controlled release and subsequent promotion of granulation tissue growth. To address such a difficult wound management system, Huang et al. [79] fabricated a sequential drug delivery system comprising an injectable hydrogel with porous poly(lactic-co-glycolic acid) or PLGA microspheres. Vancomycin was inserted into the hydrogels via the reversible Schiff’s base reaction, and recombinant human vascular endothelial growth factor 121 or VEGF were encapsulated into PLGA microspheres. The authors demonstrated that vancomycin inclusion improved the strength and elasticity of the hydrogels with shortening gelation times. Vancomycin release profiles were pH-dependent and the authors adjusted VEGF release rates by changing the pore sizes of PLGA microspheres. The duration of VEGF release was longer than vancomycin. Eventually, the authors showed that the hydrogels could inhibit bacteria growth and accelerate vein endothelial cell proliferation in vitro. In rat models, it also functioned well in managing non-healing infected wounds by reducing inflammation while promoting angiogenesis. To address the problem of tissue wounds, researchers [80,81] produced bilayer constructs for sequential release and delivery of a cutaneous antiseptics or antibiotics such as ciprofloxacin (Cipro) for wound dressing applications. In the work of Contardi et al. [80], the first layer for direct wound contact was polyvinylpyrrolidone (PVP) containing the antiseptic, Neomercurocromo^®^ (Neo, an eosin-based liquid antiseptic), while the second layer was a blend of HA and PVP containing the antibiotic. The bilayer films were shown to have satisfactory self-adhering characteristics to human skin and that PVP and HA interacted via hydrogen bonds causing sustained release of the antibiotic over a period of 5 days (see Figure 11). They evaluated the antibacterial activity of the materials against *Staphylococcus aureus*, *Escherichia coli* and *Pseudomonas aeruginosa* while the wound resorption tests were done with an in vivo full-thickness excisional wound- healing mice model.

Polystyrene sulfonates (PSS) are known to have antiseptic and antiviral properties [82]. Tomer et al. [83] demonstrated that PSS and tyrosine could be incorporated in cross-linked HA for controlled release using electric field actuation. The noted that cross-linked HA hydrogels are quickly swollen in water, but they lose much of their water content when transferred to solutions of high ionic strength. Application of an electrical field to the gels causes rapid de-swelling because of partial protonation of the ionized polyelectrolyte network. The authors loaded the HA hydrogels with high negative charge PSS and tyrosine [63]. The loaded gels got swollen in water but applying an electrical field dramatically reduced the swelling process. They argued that such a response could form the basis of a responsive and pulsatile release system for charged antiseptics or medicines, releasing them when the electric field is removed, and inhibiting release when the field is reapplied. Pitarresi et al. [84] fabricated hydrogels from polylactic acid-modified HA with or without polyethylene glycol chains. The gels were loaded with vancomycin and tobramycin and applied as coatings to titanium disks for orthopedic prosthesis. They studied in vitro antiseptic release in simulated physiological fluids as a function of drug concentrations. They also performed tests by inserting a hydrogel-coated prosthesis in a seat of a lyophilized human femur, to confirm the ability of the hydrogel to adhere to the prosthesis surface after insertion. Controlled release of antiseptics up to three days could be achieved depending on the modified HA polymer structure and drug loading. Palumbo et al. [85] produced HA copolymers from polymethacrylate groups to be used as pH sensitive release of vancomycin when transformed into hydrogels to prevent *Staphylococcus aureus* colonization. The copolymer was obtained from atom transfer radical polymerization having chargeable (carboxyl and amino) groups and formulated as a hydrogel. More specifically, it was known as HA-thylenediamine tetrabutylammonium 2-bromo-2-methylpropionic-methacrylate sodium salt copolymer. The hydrogels were produced at 5, 6 and 7 pH values with or without vancomycin (2% *w*/*v*). The vancomycin release profiles were correlated to the starting hydrogel pH values. They demonstrated that the gels could sustain the release of drug for more than 48 h. *S. aureus* adhesion tests were performed on glass culture plates and hydroxyapatite doped titanium surfaces, with similar formulations using pristine HA. They found that these new class of modified HA hydrogels prevented bacterial adhesion even without vancomycin.

Montanari et al. [86] constructed nano-hydrogels for levofloxacin (LVF) delivery to treat intracellular bacterial infections. They did not explicitly conduct release tests, but their results showed that LVF, a fluoroquinolone antibiotic, was entrapped within the gels by nanoprecipitation resulting in a drug delivery system. The nanoscale hydrogels were obtained by self-assembling of the HA-cholesterol amphiphilic chains in aqueous solutions. The minimum inhibitory concentration values of LVF-loaded hydrogels were measured for *S. aureus* and *Ps. aeruginosa* strains. Moreover, the authors also measured intracellular antimicrobial activity of the drug-loaded hydrogels on HeLa epithelial cell line infected by the abovementioned bacteria with very promising antibacterial results.

Layer by layer (LbL) assembly of HA and chitosan as coatings on activated surface of polyethylene terephthalate in order to obtain polyelectrolyte multilayers (PEMs) was implemented by Pérez-Álvarez et al. [87]. The coatings were developed to combine adhesion resistance, contact killing properties and controlled release of antibacterial agents. PEMs were loaded with triclosan (TRI) and rifampicin (RIF). The release of these bactericides could be tuned by the authors using sequential incorporation along the polysaccharide layers. HA/CHI multilayers inhibited *E. coli* adhesion and upon TRI and RIF loading, better antibacterial activity.

**Figure 12:**
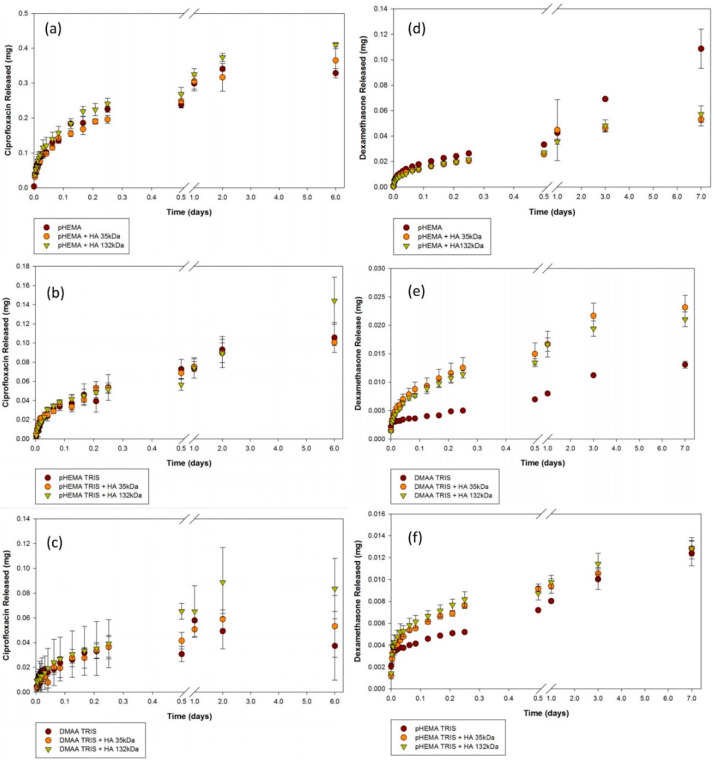
Ciprofloxacin release curves from (**a**) pHEMA, (**b**) pHEMA TRIS and (**c**) DMAA TRIS materials. Values plotted are means ± SD. HA 35 kDa = 35 kDa hyaluronic Acid. HA 132 kDa = hyaluronic Acid 132 kDa. Dexamethasone phosphate release curves from (**d**) pHEMA, (**e**) pHEMA TRIS and (**f**) DMAA TRIS materials. Values plotted are means ± SD. HA 35 kDa = 35 kDa Hyaluronic Acid. HA 132 kDa = hyaluronic Acid 132 kDa. From [88].

Nguyen et al. [88] attempted to construct contact lenses by covalent incorporation of HA into conventional hydrogels containing silicone. The lenses were loaded with antibiotic ciprofloxacin and the anti-inflammatory steroid dexamethasone phosphate. Three hydrogel material samples, namely, 2-hydroxyethylmethacrylate (pHEMA), ethylene glycol dimethacrylate (EGDMA) and *N*,*N*-dimethylacrylamide (DMAA) with tris(trimethylsiloxy)silylpropylmethacrylate (TRIS) were prepared with and without the covalent incorporation of HA. Hydrogel discs were punched from a sheet of material with a uniform diameter of 5 mm. The authors measured release kinetics by placing the drug-loaded discs in saline at 34 °C in a shaking water bath. They showed that almost all the systems tested released sufficient antibiotic to be clinically relevant in an ophthalmic lens application. Silicone-modified (TRIS) hydrogels released lower amounts of drug than the conventional gels. Sustained release even after 6 days could be maintained. The antibiotic release profiles of each material system constructed by the authors are shown in Figure 12.

**Figure 13:**
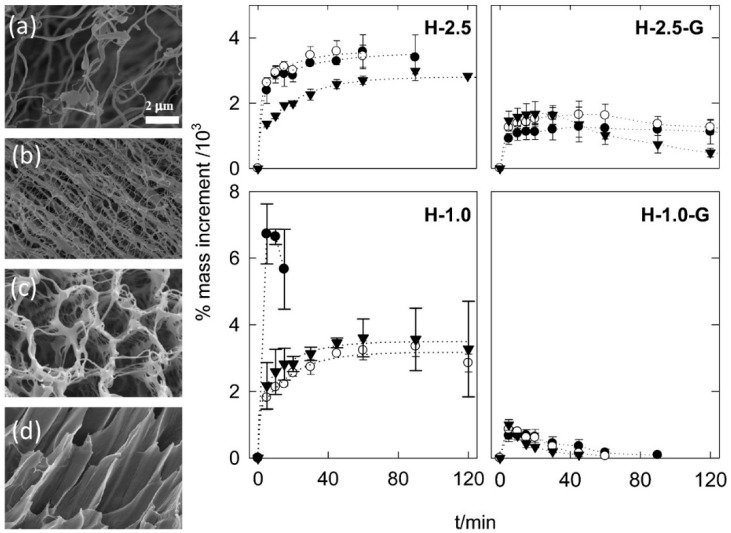
Left panel (**a**–**d**): SEM images (perpendicular to the freezing direction) of HA hydrogels produced by ice-segregation-induced self-assembly with different freezing rate (FR): H-0.1, FR = 3.6 mm min^−1^ (**a**), H-0.5, FR = 48 mm min^−1^ (**b**), H-1.0, FR = 1.8 mm min^−1^ (**c**), and H-2.5, FR = 48 mm min^−1^ (**d**). Scale bars depicted for sample (**a**) are valid for all the images. Right panel: Swelling behavior of native HA control samples containing 2.5 and 1 wt.% of HA (labeled as H-2.5 and H-1.0, respectively) and the HA samples loaded with extra content of G (labeled as H-2.5-G and H-1.0-G, respectively) frozen at 1.8 (▼), 3.6 (○), and 48 (●) mm min^−1^. G refers to Gentamicin. From [90].

Lee et al. [89] prepared a silane-modified HA hydrogel with one-pot approach using 3-glycidyloxypropyl-trimethoxysilane (GPTMS). The authors inserted the silane structure within the HA gels via self-condensation. The sol–gel-modified HA hydrogel revealed good mechanical properties and biochemical stability as well as non-toxic biocompatibility. Authors also demonstrated a drug-loading process by using sol–gel encapsulation without any additional chemicals use. Vancomycin was chosen as a model drug and it was released from the hydrogels in a prolonged manner. The first one hour was the burst release period but antibiotic release took place until 100 h. Minaberry et al. [90] constructed HA hydrogels having porous monolith structures using ice-segregation-induced self-assembly (ISISA) method and subsequent freeze drying (see Figure 13). They modified physical and chemical parameters during processing to tune porous structures and swelling characteristics in aqueous media. Gentamicin-loaded HA hydrogels were demonstrated with sustained release profiles.

SEM morphology images of the developed hydrogels via ice-segregation-induced self-assembly having different freezing rates are shown in Figure 13a–d. The right panel in Figure 13 displays hydrogel swelling and subsequent gentamicin release profiles as a function of freezing rates and HA percentages designated by H in the figure. For matrices with 2.5 wt.% HA, gentamicin release was favored for all freezing rates as seen in the figure.

Hyaluronidases are a family of enzymes that catalyze the degradation of hyaluronic acid (HA). They can be used to tune release of antimicrobials from HA systems. Ran et al. [91] recently demonstrated such an action. They constructed a hyaluronidase-triggered photothermal material for killing bacteria based on silver nanoparticles (AgNPs) and graphene oxide (GO). AgNPs and GO were embedded in HA polymer forming nanocomposites. Hyaluronidase (HAase)-triggered release of HA coated AgNPs displayed antibacterial activity against *Staphylococcus aureus*. Upon illumination the nanocomposites with near infrared light, GO locally raised the temperature and with the action of HAase, AgNPs were released. Presence of HA over the surface of the nanoparticles did not created any toxicity to mammalian cells. They also tested their material in a wound disinfection model with encouraging results. Zhang et al. [92] designed long-lasting antibiotic release systems for postoperative infection elimination in ophthalmic surgery. Ciprofloxacin and vancomycin-conjugated hyaluronic acid HA were synthesized for sustained release of antibiotics (up to 200 h). The authors also measured antimicrobial effects of the released drugs by disc-diffusion and macro-dilution tests at different times up to 2 weeks. The amounts of drugs loaded and the degradation rate of HA particles were controlled to obtain long-term release profiles that can inhibit the growth of bacteria for up to one week, suitable for postoperative infection prevention in ophthalmic surgery. Lequeux et al. [93] demonstrated that nisin (an antimicrobial peptide) could be attached to HA to obtain antimicrobial gels. Various amounts of nisin were grafted onto HA via controlled reactions forming covalent bonds among amide groups. The antimicrobial activity of the modified HA was tested against *S. epidermidis*, *S. aureus* and *Ps. aeruginosa* bacteria. In solution, modified HA exhibited a great antimicrobial property on the three tested bacterial species. Although they did not measure sustained release of nisin they found that within a 24 h period effective bacterial free zones were formed around the gel-like films prepared by the authors. Similarly, Silva et al. [94] encapsulated antimicrobial peptides (AMPs) in HA nanogels to kill mycobacteria responsible for Tuberculosis (TB), a disease caused by the human pathogen *Mycobacterium tuberculosis*. The specific AMP was called AMP LLKKK18. The authors showed that encapsulation into HA provided increased stability, reduced cytotoxicity and degradability, while potentiating peptide targeting to main sites of infection. In vitro tests with the opportunistic *M. avium* or the pathogenic *M. tuberculosis*, showed lowered pro-inflammatory cytokine levels (IL-6 and TNF-α) in a sustained way within a 72 h period. Further work by authors on mice demonstrated significantly reduced infection levels due to with *M. avium* or *M. tuberculosis*, after just 5 or 10 every other day administrations. Park et al. [95] produced 1-ethyl-(3-3-dimethylaminopropyl) carbodiimide hydrochloride-cross-linked collagen- HA polymeric matrices loaded with tobramycin or ciprofloxacin antibiotics targeting wound contamination. In vitro release experiments demonstrated that tobramycin and ciprofloxacin loaded matrices maintained their antibacterial effects for over 96 and 48 h, respectively. Up to 90% release could be achieved within this period. They also incorporated growth factors in their modified HA formulations and observed significant wound healing in in vivo full thickness dermal defect model. They argued that their materials can be eventually designed as skin replacements for severely infected skin diseases. Luo et al. [96] developed a novel HA-based hydrogel and evaluated for sustained drug release. They showed that the gels cross-linked in minutes and the dried forms swelled and rehydrated to a flexible hydrogel in seconds. HA was first transformed into adipic dihydrazide derivative and then cross-linked with poly(ethylene glycol)–propiondialdehyde forming a polymer network. After solvent casting, the authors dried the films and allowed them to swell sevenfold in volume in buffer, in less than 100 s. Sustained drug release from the hydrogel films was evaluated in vitro using selected anti-bacterial and anti-inflammatory drugs such as diclofenac sodium, pilocarpine, acceptindomethacin, hydrocortisone, 6α-methylprednisolone, prednisolone, cortisone, corticosterone, dexamethasone and prednisone. They were probably the only ones who have measured release profiles so many drugs from a single HA-based gel. All drugs were measured for a period of 100 h and about 80% of the drugs were released with the first 40 h period.

Finally, Table 2 summarizes the sustained release systems reviewed in this section. The table shows that mostly, hydrogels have been developed but also films and multilayer constructs appear to be effective. Most studies demonstrated sustained release periods exceeding a few days. This is important particularly for skin wound management treatments. A majority of the targeted therapies focused on soft tissue and cutaneous treatments but also eye-related diseases. As Table 2 demonstrates, frequently used model antibiotics are the ones that are commercially used such as ciprofloxacin, vancomycin and levofloxacin etc. Very few others are based on peptides and natural extracts like catechins. As matrices of choice, Table 2 displays that polymeric adducts or blends of HA were extensively used to induce sustained release. HA polymer by itself is known to have therapeutic potential in the treatment of arthritis and wound healing [97]. Hence, inclusion of small quantities of antibiotics in HA-based matrices could be very effective local treatment means since they will potentially reduce the oral antibiotic intakes and related side effects.

## 5. Hyaluronic Acid and Cancer Targeting Drugs

HA has been widely used in anticancer drugs delivery due to its excellent biocompatibility, biodegradability and specific targeting to cancer cells. It is now acknowledged that to improve the efficiency of cancer cell delivery processes, HA-based matrices need to become “transformers”. A very recent study showed that HA based matrices are among the very few biopolymers that can satisfy the “3S” transition approach for anticancer drugs [98]. The “3S” transition concept is known as stability transition, surface transition and size transition and if all these three concepts are satisfied in drug delivery systems, all the barriers in delivery processes are overcome and the drug works. More specifically, stability transition refers to when the drug enters and circulates in vivo, the matrix-drug system needs to be stable and minimize the systemic toxicity caused by the early release phase of the drug. The surface transition refers to the transition of drugs (during release) from a smooth solid surface to uneven and viscous surfaces that are accompanied by surface charge changes and other adsorbed agents such as enzymes that can degrade the matrix surface rapidly inferring with drug release. The size transition is directly relevant to tumors. Size is associated with affecting half-life in vivo and accumulation of tumor tissue. In general, vascular tissue near the tumor tissue has poor structural integrity with a missing lymphatic reflux system. As such, small/nanoscale matrix-drugs could be deeply retained near the tumor tissue. This is known as enhanced permeability and retention (EPR) effect. They are also susceptible to easily removal by blood flow due to their size. It is therefore argued that in order to enhance the EPR effect, HA-based matrices need to be large in size in the blood circulation, but must be under internal or external stimuli so that their size can reduce near the tumor tissue such as by the action of hyaluronidase. Hence, HA is a unique matrix for cancer targeting drug release since its size can be modulated in vivo with hyaluronidase enzymes.

HA and its derivatives can specifically bind to various receptors on the cell surface and can be used for targeted drug delivery of anti-tumor drugs. Hence, several such tumor-targeted drug delivery systems based on HA have been developed so far. Huang and Huang [99] published a review of advances in tumor targeting drug release from HA. This section will supplement and somewhat extend their work. Dosio et al. [100] reviewed anticancer material systems containing HA. They focused on imperative in vivo studies for estimating the clinical feasibility of drug delivery systems. Their review included several approaches on published preclinical/clinical data. An earlier review by Jaracz et al. [101] presented advances in tumor-targeting drug conjugates including HA as tumor-targeting moiety. They highlighted the fact that HA level is elevated in various cancer cells. As the concentration of HA in cancer cells increases, cells tend to form a less dense matrix, thus enhancing cell motility as well as invasive ability into other tissues. HA is also known to form an immune protective coat against cancer cells. For instance, various tumors like epithelial, ovarian, colon, stomach, and acute leukemia, overexpress HA-binding receptors CD44 (cell-surface glycoprotein involved in cell–cell interactions) and receptor for HA-mediated motility (RHAMM). Both reviews did not discuss sustained or controlled release of anticancer drug aspects but rather focused on approaches that would minimize side effects related to chemotherapy.

**Figure 14:**
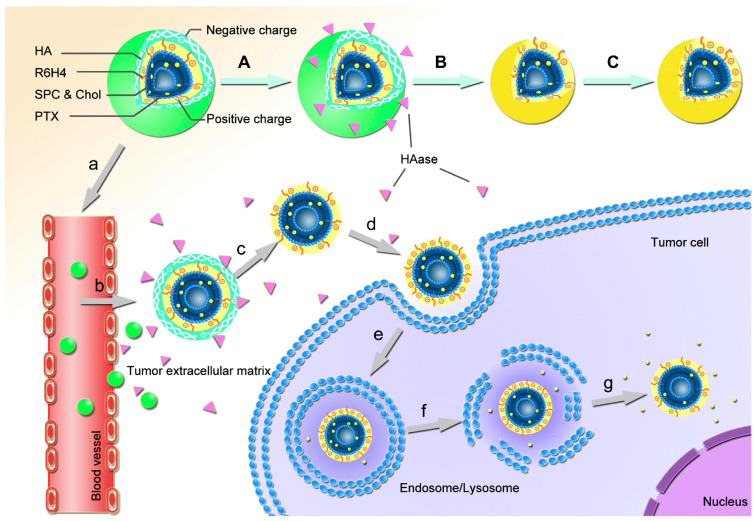
Schematic design of the dual-decorated liposomes (PTX/HA-R6H4-L) for tumor-targeted drug delivery. R6H4 is a cell penetrating peptide. PTX: paclitaxel, SPC: Soy phosphatidylcholine and Chol: Cholesterol A—Degradation of HA by HAase. B—Disassembly of PTX/HA-R6H4-L to PTX/R6H4-L. C—Tumor pH-induced PTX/R6H4-L for enhanced cell penetration. a—Intravenous injection of liposomes. b—Accumulation of liposomes at the tumor site through passive and active targeting. c—Degradation of HA by HAase together with the exposure of R6H4 at HAase-rich tumor milieu. d—pH-Response of R6H4 to mildly acidic tumor microenvironment for improved cellular uptake. e—Internalization of liposomes into the tumor cells. f—Endosomal/lysosomal escape. g—Cytoplasmic release. From [102].

Jiang et al. [102] combined pH-responsive cell-penetrating peptides (CPPs) effective for intracellular delivery with HA having good blood persistence for tumor targeting. They developed dual-decorated liposomes for tumor-targeted drug delivery (Figure 14).

They showed that in blood stream HA-coated CPP-modified liposomes (HA-CPP-L) possessed strongly negative charge and protected by plasma protein attack by HA shell for enhanced stability and duration. HA-CPP-L demonstrated high accumulation at the tumor site due to enhanced permeability and retention and the affinity of HA with binding receptors. The authors were able to demonstrate that at the tumor milieu, HA-CPP-L disintegrated to CPP-L HAase enzyme degradation of HA. As a result, the exposed CPP responded to the mildly acidic tumor microenvironment to increase the uptake of CPP-L into the cells. They measured the effect of CPP release into the cell by monitoring the tumor volume changes as a function of time against control tests in mice (Heps tumor xenograft models). A sustained decline in tumor size throughout a 16-day period was confirmed by the authors [102]. Similarly, Miao et al. [103] also observed sustained decrease in tumor volume in mice models when they encapsulated doxorubicin (Dox) in HA-modified reduced graphene oxide (rGO) nanosheets (a few layer graphene agglomerates, 3–6 nm in thickness).

The nanosheets were prepared by coating cholesteryl hyaluronic acid (CHA) on rGO nanosheets. They displayed a survival rate of 100% after intravenous administration of 40 mg/kg in mice. Uptake of Dox by CD44-overexpressing KB cells was higher for CHA-rGO than for rGO. Note that KB cells are known to be a subline of the abundant keratin-forming tumor cell line HeLa (standard human lab-test cell lines). After intravenous administration in tumor-bearing mice, CHA-rGO/Dox displayed higher tumor accumulation than rGO/Dox facilitating the cellular uptake of Dox by CD44-overexpressing tumor cells with enhanced anticancer effects. The sustained cellular delivery effect of released Dox from HA rGO sheets was monitored for 24-day period by measuring the tumor volume decline.

Nanohybrid liposomes coated with amphiphilic HA–ceramide (HACE) was fabricated by Park et al. [104]. Nanohybrid liposomes in 120–130 nm mean diameter contained DOX and Magnevist, a contrast agent for magnetic resonance (MR) imaging. DOX release from the nanoparticles was improved under acidic pH (pH 5.5 and 6.8) levels versus physiological pH (pH 7.4). The authors showed that cellular uptake of DOX from the nanohybrid liposome was enhanced by HA and CD44 receptor interaction, versus the plain liposome. DOX release could be sustained up to 160 h in in vitro tests with higher percent drug release at lower pH values.

**Figure 15:**
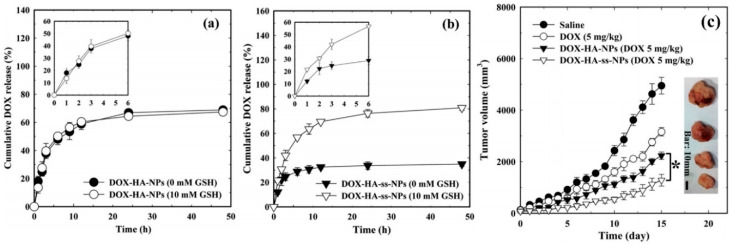
In vitro release behavior of DOX from HA nanoparticles in the presence and absence of GSH: (**a**) DOX-HA-NPs and (**b**) DOX-HA-ssNPs. Insets are magnified drug release graphs (0–6 h). The error bars in the graph represent standard deviations (*n* = 3). (**c**) Tumor growth of SCC7 cancer xenografts treated with saline, free DOX, DOX-HA-NPs, and DOX-HA-ss-NPs at a DOX dose of 5 mg/kg. Insets are representative tumor images excised 16 days post-treatment. Error bars in the graph represent the standard deviation for five animals per group. Asterisks (*) denote statistically significant differences (*p* < 0.05) as determined by one-way ANOVA. From [105].

Han et al. [105] developed tumor-targeted nano drug carriers based on HA-polycaprolactone (PCL) block copolymer where HA was cross-linked by a disulfide linkage. The anticancer drug DOX was also utilized by the authors. It was encapsulated in the disulfide crosslinked HA-PCL nanoparticles (DOX-HA-ss-NPs). Drug release under physiological conditions (pH 7.4) was very slow, but the DOX release rate was markedly enhanced in the presence of glutathione (GSH, a thiol-containing tripeptide) that can breakdown disulfide bonds in the cytoplasm. The authors also showed that DOX-HA-ss-NPs could easily deliver DOX into the nuclei of SCC7 cells in vitro as well as to tumors in vivo in tumor-bearing mice. Figure 15a shows that DOX release rates are practically similar for the copolymer regardless of GSH presence in the release medium. However, in the case of disulfide cross-linked system, GSH enhances DOX release almost twice as seen in Figure 15b. The same system also enables the smallest tumor volume development on mice experiments as displayed in Figure 15c. A recent review by Kim et al. [106] also reviewed HA coated nanomaterials that have been developed for targeted cancer therapy but they did not discuss any aspects related to controlled or sustained drug release. Similarly, Snetkov et al. [107] reviewed recent advances in HA-based nanofiber fabrication protocols as well as their biomedical applications with cancer therapy. They focused on production of nano- and microfibers from HA by utilizing other biocompatible medical polymers such as polyethylene oxide (PEO) and polyvinyl alcohol (PVA) including binary/ternary solvent systems rather than their drug release characteristics.

DOX-loaded HA-based nanoparticles (NPs) made by interconnected hyaluronic acid-ceramide (HACE) and their in vitro release kinetics were reported by Park et al. [108]. Interconnected HACE was synthesized by cross-linking HACE with adipic acid dihydrazide (ADH) leading to NPs of the order of 200 nm in size. Interconnected HACE-based NPs displayed sustained drug release with more efficient drug encapsulation compared to HACE-based NPs. Release times as long as 160 h were observed with up to 80% encapsulated DOX release depending in the pH. More release was monitored under lower pH conditions (pH = 5.5) indicating the possibility of enhancing drug release in tumor region and endosomal compartments. Xin et al. [109] developed a cell-targeted prodrug with self-assembly properties in aqueous solution to encapsulate anti-cancer drug paclitaxel. They combined and conjugated paclitaxel with HA by inserting different amino acids as spacers, including valine, leucine, and phenylalanine, respectively. These prodrugs were shown to self-assemble to form nanoparticles and their in vitro drug release kinetics were monitored for up to 100 h under different pH conditions. Depending on the amino acid type, drug release could be controlled from about 20% to 90% with different release kinetics under acidic or neutral pH levels. Another prodrug was prepared from taxol and chemically modified HA by Luo et al. [110]. They synthesized a fluorescent labeled HA-taxol (FITC-HA-taxol) conjugate and demonstrated cell-specific binding and uptake. They studied the in vitro release of taxol from the conjugates in human plasma or in cell culture media. They argued that the release was possible through cleavage of the ester linkages within the conjugates. Sustained release in human plasma was about 40% within the first 30 h of the measurements. This was further increased to 80% levels with the same period by using hyaluronidase enzyme in the same media that the authors used.

**Figure 16:**
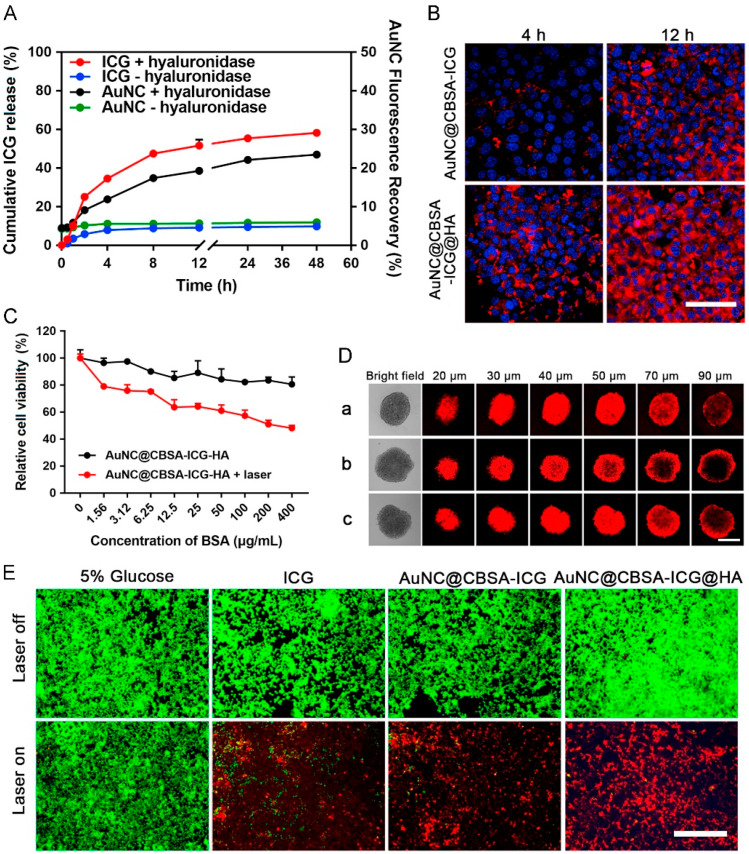
(**A**) Cumulative ICG release and fluorescence recovery (drug release real time imaging) with or without hyaluronidase through release procedure. (**B**) Cellular uptake of nanoparticles after incubation for 4 and 12 h. The white bar represents 50 μm. (**C**) Cell viability measured by MTT assays. (**D**) Nanoparticles penetrating into tumor spheroids. The white bar represents 200 μm, a—represents AuNC@CBSA-ICG, b—represents AuNC@CBSA-ICG@HA and c—represents AuNC@CBSA-ICG@HA pretreated with hyaluronidase. (**E**) The in vitro photothermal effect of formulations measured by calcein-AM/PI double staining. The white bar represents 200 μm. From [113].

Nanoscale HA systems appear to be very effective tumor targeting vehicles particularly if they are tailored with controlled drug release means. They can be as effective as and with even more in vivo success compared to other nanoparticle tumor targeting vehicles [111,112]. Liu et al. [113] fabricated cationic small-sized red emission BSA-protected gold nanocluster (AuNC@CBSA, 21.06 nm) in order to encapsulate indocyanine green (ICG) as imaging agent and also to realize theranostic treatment. Following this, AuNC@CBSA-ICG systems were complexed to negatively charged HA to form nanoparticles of about 200 nm in size (AuNC@CBSA-ICG@HA). HA-modified nanoparticles could be degraded by hyaluronidase into small nanoparticles that could penetrate into tumors also enabling sustained drug release for potential for breast cancer therapy. To study in vitro release rates the fluorescent rate was fitted with the ICG dose by an exponential function (not shown for brevity), that was used to estimate ICG release amounts. Upon contact with hyaluronidase the ICG release measurements indicated the cumulative release was elevated over 60% of total drug loading after 48 h release (see Figure 16). Similarly, the fluorescence recovery curve agreed with the release data confirming drug release in real-time. Note that fluorescence recovery is a method for determining the kinetics of diffusion through tissue or cells. It is capable of quantifying the two-dimensional lateral diffusion of fluorescently labeled probes into biological environment. However, without hyaluronidase, less than 10% ICG was released after 48 h period. The low growth in the fluorescence recovery curve also confirmed this.

In a unique study, Gurski et al. [114] reported HA-based hydrogels encapsulating cancer cells for drug delivery. The hydrogels supported cell growth and viability in which cancer cells (prostate cancer cells, C4-2B) adopted a rounded, clustered morphology similar to tumor tissue in vivo. Drugs were released into the HA hydrogel to kill cells and the HA hydrogel was studied for drug (camptothecin, docetaxel, and rapamycin and their combinations) dose and time dependence. Sustained drug release in the hydrogels for a period of 24 h was achieved with no cell toxicity. The HA-based hydrogels were made up of HA derivatives having aldehyde and hydrazide pendant groups [114]. Sun et al. [115] constructed a light-activated nanoscale HA-based drug delivery systems with on-demand drug release at tumor sites. Structures encapsulating DOX were self-assembled from a HA-photosensitizer conjugate containing reactive oxygen species (ROS)-sensitive thioketal (TK) linkers. After encapsulating the drug, HA-based nano-carriers were accumulated in the MDA-MB-231 breast tumor xenograft. The accumulation prevented drug leakage in the bloodstream. A 660 nm laser irradiation cleaved the TK linkers resulting in light-induced material dissociation and selective DOX release in the tumor area, minimizing toxicity in vivo. Up to 70% cumulative release within 50 h period could be possible with their system.

**Figure 17:**
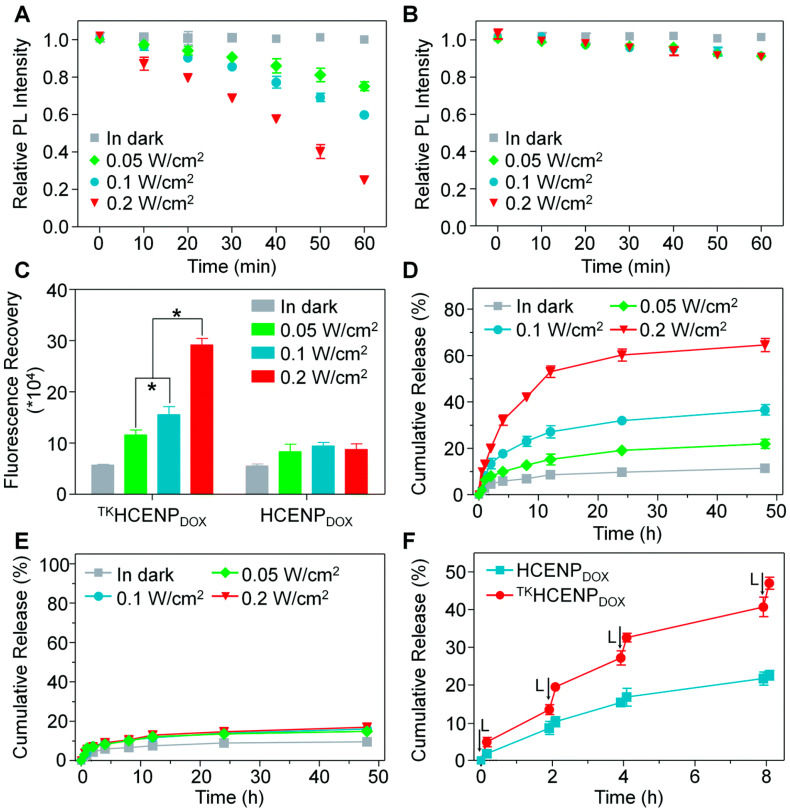
Fluorescence quenching of NR from ^TK^HCENP_NR_ (**A**) and HCENP_NR_ (**B**) in aqueous solution after light stimulation at pH 7.4. (**C**) Fluorescence recovery of DOX from ^TK^HCENP_DOX_ and HCENP_DOX_ after light stimulation at pH 7.4. The cumulative release of DOX from ^TK^HCENP_DOX_ (**D**) and HCENP_DOX_ (**E**) upon 660 nm laser irradiation at different power densities for 30 min. (**F**) Laser-stimulated pulsed release of DOX from HCENP_DOX_ and ^TK^HCENP_DOX_. The samples were irradiated with a 660 nm laser (0.2 W cm^−2^) for 10 min at different time points indicated by the arrows. ^TK^HCENP_NR_ refers to light activated nano-carrier with thioketal linkers containing Nile red dye and ^TK^HCENP_NR_ refers to without thioketal linkers containing Nile red. ^TK^HCENP_DOX_ refers to the same as before containing DOX drug. From [115].

Figure 17A,B show that in dark, no significant quenching was monitored in both nano-carrier systems. However, thioketal-linked HA system could quench as a function of radiation intensity as seen in Figure 17A, compared to the thioketal free HA-based carriers, with very little fluorescence quenching. The quenching recovery was also superior for thioketal-linked HA system (Figure 17C). Quenching also triggered DOX release from the thioketal linked-HA carriers as seen in Figure 17D compared to thioketal free HA nanocarriers (Figure 17E). Pulsing the 600 nm laser also induced spikes in release profiles during release as seen in Figure 17F.

**Scheme 3:**
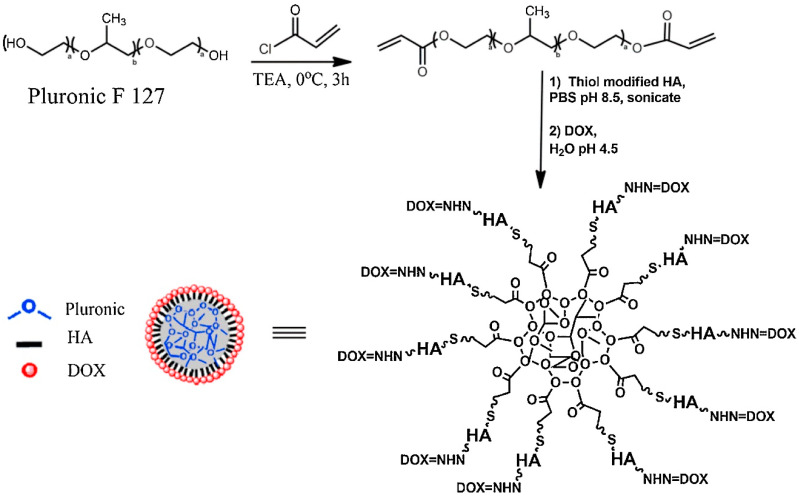
Schematic of chemical synthesis process of Poloxamer 407-modified HA nanogels. From [118].

So far HA has been widely implemented as a favorable biopolymer for producing advanced clinical cancer therapies in various forms such as nanoparticles, micelles, liposomes, and hydrogels, combined with other materials [116]. However, as can be seen from this section, a fraction of such studies reported sustained drug release data. A prodrug gelation strategy was developed by Fu et al. [117] for sustained release of dual stimuli-response cancer drug known as doxorubicin hydrochloride (DOX·HCl), DOX·HCl was chemically conjugated onto thiolated HA by an acid liable hydrazone linkage. Upon air exposure, the conjugate gelled in aqueous solution. Thiol groups could tune the gelation time and extent on HA. The hydrogel released conjugated DOX·HCl in a sustained manner in dual pH- and reduction-responsive modes. The cumulative drug release was much faster under the conditions mimicking the intracellular environments of cancer cells. The in vitro cytotoxicity assays for the human nasopharyngeal carcinoma CNE2 cells confirmed the efficacy of the conjugate hydrogels for cancer cell inhibition [117]. Similarly, Gurav et al. [118] prepared HA nanogels (150 nm in diameter) with thiol modified HA and diacrylated Poloxamer 407 polymer. Using a Michael type addition reaction (Scheme 3) of activated thiol groups on acrylate moiety simply formed the nanogels. The nanogels also encapsulated DOX for evaluating sustained drug release properties. Nanogels prepared were of 150 nm in diameter with a narrow size distribution pattern. DOX released from these nanogels showed a slow and sustained release at acidic pH 5.0 whereas very little release was measured at physiological pH 7.4. The sustained drug delivery system continued DOX release after 24 h without affecting normal cells. The authors argued that these nanogels could deliver anticancer drugs without impeding their toxicity value over longer durations and reducing the total dose amount in anticancer therapy.

**Figure 18:**
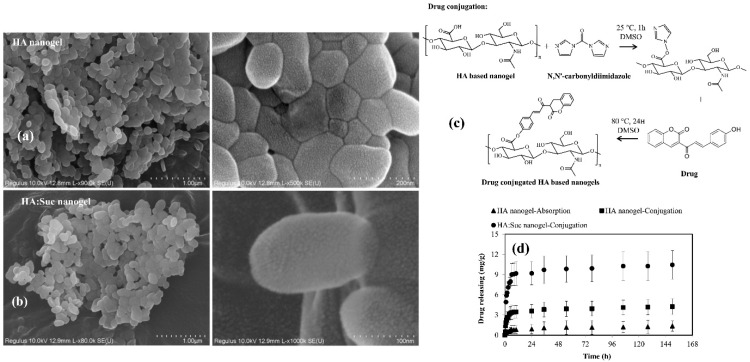
SEM images of (**a**) HA and (**b**) copolymeric HA/sucrose Suc nanogels. (**c**) Schematic representation of drug conjugation process to HA-based nanogels, and (**d**) Drug release profiles from drug-absorbed and -conjugated HA nanogels and drug-conjugated HA:Suc (HA/sucrose) nanogels at physiological condition at pH 7.4 buffer solution containing 5% DMSO solution at 37.5 °C. From [122].

Yin et al. [119] developed, a redox-sensitive, HA-decorated GO nanosheet for tumor cytoplasm-specific rapid delivery using near-infrared (NIR) irradiation-controlled endo/lysosome disruption and redox-triggered cytoplasmic drug release. HA modification through redox-sensitive linkages allowed graphene oxide particles to attain certain extraordinary attributes such as biological stability, enhanced drug-loading capacity for aromatic molecules, HA receptor-mediated active tumor targeting, greater NIR absorption and thermal energy translation, and a sharp redox-dependent response for faster drug release. They also used DOX as a model drug and cytoplasm-specific DOX release was achieved via NIR controlled endo/lysosome disruption along with redox-triggered release of DOX in glutathione rich areas. Up to 70% cumulative DOX release within the first 16 h of measurements was reported by the authors. HA appears to be at the forefront of studies on development of injectable and biocompatible vehicles for delivery, retention, growth, and differentiation of cancer drugs as well as stem cells for cell therapy. It is now very important to ensure that more clinical combination products are developed using HA with highly reproducible, scale-up suitable, approvable, and affordable means [120]. Seok et al. [121] developed HA cross-linked zein nanogels to deliver curcumin, a naturally occurring phytochemical drug in cancer cells. Curcumin encapsulated HA-zein nanogels displayed high anticancer activity against the CT26 cells. The in vivo bio-distribution in CT26 tumor model exhibited high tumor accumulation, improving antitumor efficacy with a low curcumin dosage. The encapsulated curcumin was effective for a 15-day period indicated by tumor volume reduction by about five times compared to control tests. Suner et al. [122] fabricated porous HA nanogels copolymers with sucrose (Suc). Nanogels (about 200 nm in size), were synthesized by engaging glycerol diglycidyl ether (GDE) as cross-linker with a single step reaction (Figure 18). The authors showed that the nanogels were stable in blood up to 250 μg/mL concentration with approximately 0.5 ± 0.1% hemolysis ratio and 76 ± 12% blood clotting indices, respectively. They also demonstrated that the nanogels could encapsulate and slowly release a hydrophobic cancer drug, 3-((E)-3-(4-hydroxyphenyl)acryloyl)-*2H*-chromen-2-on (A^#^) for up to two days.

In summary, Huang and Huang [99] indicated that tumor-targeted drug delivery systems based on HA are classified into four approaches. HA-drug conjugates, HA amphiphilic derivatives, HA surface modifications, and HA-gene drugs, respectively. However, they stressed that most of these works are still somewhat far from clinical uses and that such more HA systems must be developed towards immunotherapy. The function of HA depends on its size, location, and interactions with its binding patterns. The interactions of HA and its binding proteins contribute to the pathogenesis of several diseases including cancer. Thus, new sustained release materials based on HA and the study of their interactions with cells and proteins may provide new approaches to developing therapeutics for inflammatory and fibrosing diseases [123]. Finally, Table 3 below summarizes the type of drugs, matrices, tumor test platforms as well as the drug release periods extracted from the studies reviewed in this section. The table indicates that DOX is the most studied drug on various mice models. Moreover, instead of macroscopic hydrogels, HA systems were in general formulated as nanoscale matrices such as nanogels or nanoparticles. This is in accordance with the fact that such tumor targeting drugs should permeate cell walls and membranes. As the controlled release matrix, very few works used pure HA and the rest were either HA conjugates, copolymers or blends with other natural polymers such as sucrose and zein as seen in Table 3. It appears that each HA matrix system was developed for a specific cancer drug but more universal HA-based matrices that can release a variety of cancer medicines would be very beneficial for tumor targeting treatments. Today, only about one out of every twenty published studies focuses on encapsulating and controlled release of various anti-cancer drugs from a common HA-based matrix. More work needs to be channeled toward this goal to unite and simply cancer and tumor targeting studies.

## 6. Summary and Perspectives

The aim of this review was to demonstrate and present recent advances in controlled release of various drugs from HA-based matrices but also the release of HA itself from other biomaterials particularly suited for soft tissue treatments. As demonstrated, the sustained release systems can be produced as films, macro/hydrogels, polymeric microparticles, nanogels, nanoparticles but also as multilayer films. Inspecting Table 1, Table 2 and Table 3 indicates that drug release periods up to one month and in certain cases near two months were measured and as such, one can conclude that the developed HA-based matrices were stable in both in vitro and in vivo environments. Moreover, it has been demonstrated that HA can be cross-linked into several robust polymeric networks that can delay its erosion and dissolution in aqueous media. For instance, HA can be cross-linked with water-soluble carbodiimides, glutaraldehydes, chitosan, collagen, thiols, methacrylates (photo-cross-linking), divinyl sulfones, epoxied poly(ethyelene glycol)s, enzyme/amine grafts, hydrazide grafting. The readers can refer to Khunmanee et al. [124], which is a recent review on crosslinking methods of HA-based hydrogels for biomedical applications. HA can also be blended with other water-soluble biopolymers such gelatins, alginates, chitosan, cellulose derivatives, polyvinyl alcohols to name a few. The aim of such works appears to be extension of drug release periods from HA-based matrices.

Secondly, HA is an intrinsic therapeutic agent. It is an excellent lubricant for the eyes and the bone joints. For this reason, the state-of-the-art related to sustained delivery of HA from other biomaterials/biopolymers were also reviewed. To date, most studies focused on release and delivery of HA from soft contact lenses, artificial joint gels and hydrogel films for lubrication. Continually lubricated eyes are crucial for eye health and for the growth of cartilage and bone. Therefore, it is important to develop soft biomaterials that can render joint tissues and eyes lubricated with minimal inflammation by delivering HA in a sustained and prolonged manner. More research and development studies on biomaterials capable of providing sustained delivery of HA and HA complexed with other important lubricants such as lubricin [14] must be conducted.

In addition, as reviewed in Section 3, protein delivery from HA-based materials has been extensively studied and new and innovative sustained release systems have been reported. For instance, efficient and controlled protein and protein-complexed molecules delivery from HA have been successfully employed to stimulate cellular processes that lead to tissue regeneration. There are, however, challenges related to coupling proteins to HA while preserving the protein’s conformation and activity [125]. Proteins such as BSA can be released in a sustainable way from HA-based hydrogels for up to 30 h without having any cytotoxicity that can meet the needs of safe cartilage repair operation practices [126]. Note that cartilage repair and regeneration is a treatment for joints that have damaged cartilage but are otherwise healthy. Their treatment generally involves filling cartilage defects by medicated biomaterials. Oh et al. [53] indicated that while commercialization of HA-based protein delivery systems still needs more work such as approvals, significant progress has been reported on HA-based protein carriers for target specific and long-acting delivery. HA polymer needs to be properly conjugated in order to bind and release proteins in certain environments. Specifically, amide, disulfide and thioester conjugations appear to be promising [53]. Improving the role of HA and HA-based biomaterials in sustained protein delivery is deemed crucial because many proteins have low bioavailability in several non-invasive treatments, a short half-life in vivo and physicochemical instability. For this reason, doctors need to use invasive administration routes by long-term infusions and/or frequent and painful injections for specific protein delivery. For example, recombinant human growth hormone (rhGH) is generally administered daily by injection over a period of several years to treat short stature in children [64].

Summarizing Section 4, one can conclude that the topical treatment of wound infections is rather challenging due to limited drug availability and delivery at local infection sites. However, it has been proven that for open wounds, local or site-specific sustained release of antiseptics and antibiotics from biomaterials is very effective in reducing systemic side effects related to much higher antibiotic dosages taken orally [127]. The unique chemical interactions of HA with other polysaccharides such as inherently antimicrobial chitosan can be used to produce very effective antiseptic materials such as wound gauzes [128]. Their healing and antiseptic properties can be further boosted by encapsulating proper antiseptics or antibiotics in such HA-chitosan hydrogels. Antibiotics such as gentamicin are very effective against bacterial infections, mostly gram-negative bacteria including *Pseudomonas*, *Proteus*, *Escherichia coli*, *Klebsiella pneumoniae*, *Enterobacter aerogenes*, *Serratia*, and the Gram-positive *Staphylococcus*. However, it has many adverse effects such as nerve and kidney damage, allergic reactions, inner ear disorders and low blood cell counts. HA-based hydrogels can be used to release gentamicin and similar antibiotics in a controlled and sustained manner for deep infections [129], increasing the efficiency but also reducing prospects of such side effects. More research efforts must be dedicated to layer-by-layer assembly of HA-based structures containing antimicrobial agents such as silver nanoparticles to form multifunctional HA-based hydrogels that can be used for biosensing and release-on-sensing [130].

Finally, Section 5 showed that HA is indispensable in structuring tissue architecture, in cell motility, in cell adhesion, and in proliferation processes. Such cellular events are facilitated mainly through two major signal-transducing cell surface HA-receptors, CD44 and the HA-mediated motility (RHAMM), designated as CD168 receptor [131]. This puts HA at the forefront of cancer- related drug delivery studies compared to other polysaccharides. Although effective cancer therapy prospects for HA have been demonstrated recently that mainly targeted prostate, gastric, breast, bladder, lung and ovarian cancers, more comprehensive studies on sustained release of tumor targeting drugs from HA and its derivatives should be conducted along with careful tumor volume reduction and drug toxicity measurements [132]. Based on such in antitumor efficacy studies, effective HA-based prodrugs can be developed for preclinical testing and evaluation. Furthermore, with increasing evidence for the expression of CD44 on cancer stem cells of diverse origins, the ability to selectively target chemotherapeutic agents to CD44 may achieve striking therapeutic success and a swift transformation into commercialization [133].

## Figures and Tables

**Table 1:** Summary of protein release systems based on HA. Please see text for protein abbreviations.

Protein Type	Targeted Therapy	Matrix System	Matrix Morphology	Release Duration	Reference
BMP-2	Cartiledge repair	Heprain modified HA	Nanoparticles	14 days	[54]
rhGH	n/a	Poloxamer 407-HA	Hydrogel	14 days	[55]
α-Amylase/lysozyme	Cutaneous Skin	Tyramine-HA	Hydrogel	2 days	[56]
siRNA	Gene Silencing	Disulfide-HA	Nanogel	120 min.	[57]
Fab1	n/a	Tetrazine-HA	Hydrogel	30 days	[58]
BSA	Tissue engineering	Methacrylate-HA	Microspheres	30 days	[59]
Rh-BMP-2	Bone regeneration	Hydrazone-HA	Hydrogel	28 days	[60]
EPO	Brain Injury	Thiolated-HA	Micro-hydrogel	9 days	[61]
BMP-2	Osteogenic	Chitosan-HA	Polyelectrolyte complex	30 days	[62]
BSA	n/a	Chitosan-HA	Polyampholyte hydrogel	21 days	[63]
Lysozyme	n/a	Dopamine-HA	Nanoparticles	n/a	[64]
rhGH	n/a	Cholesteryl-HA	Nanogel	10 days	[65]
BSA	Bone Defects	Chitosan-HA	Polyelectrolyte complex	2 days	[66]
SDF-1α	Tissue engineering	Sulfated-HA	Hydrogel	12 days	[67]
BSA	n/a	Hydrazide-HA	Hydrogel	10 days	[68]

**Table 2:** Summary of antiseptic/antibiotic release systems based on HA. Please see text and cited references for abbreviations.

Drug Type	Targeted Therapy	Matrix System	Matrix Morphology	Release Duration	Reference
Epigallocathecin	Tendinopathy	Adipic acid-HA	Hydrogel	10 days	[74]
Gentamicin	Osteosynthesis implants	Acrylamide-HA	Gel	7 days	[75]
Various	Osteosynthesis fracure	Polylactide-HA	Hydrogel	n/a	[76]
Triclosan	Medical implants	Chitosan-HA	Multilayers	8 days	[77]
CHX	Hemorrage control	PEG-HA	Hydrogel	10 days	[78]
Vancomycin	Infected wounds	Chitosan-HA	Microsphere hydrogels	20 days	[79]
Ciprofloxacin/Eosin	Skin wounds	PVP-HA	Multilayers	5 days	[80]
Chitosan	Tissue engineering	Chitosan-HA	Multilayers	40 days	[81]
PSS	n/a	HA	Gel	9 days	[83]
Vancomycin/Tobramycin	Orthopedic prosthesis	PLA-HA	Hydrogel	3 days	[84]
Vancomycin	Biofilm infections	Methacrylate-HA	Hydogel	2 days	[85]
Levofloxacin	Intercellular Infection	Cholestrol-HA	Nanogel	7 days	[86]
Triclosan/Rifampicin	Post implant infection	Chitosan-HA	Multilayers	25 days	[87]
Ciprofloxacin/Dexamethasone phosphate	Contact lens infection	Silicone-HA	Hydrogel	6 days	[88]
Vancomysin	n/a	Silane-HA	Hydrogel	4 days	[89]
Gentamicin	Soft tissue scaffolds	HA	Porous hydrogels	4 h	[90]
Ag NPs	Synergystic therapy	rGO-HA	Nanocomposite	n/a	[91]
Vancomycin/Ciprofloxacin	Opthalmic surgery	Diglycid ether-HA	Microparticles	8 days	[92]
Nisin (petide)	Contact lens infection	HA	Films	1 day	[93]
LLKKK18	Tuberculosis	HA	Nanogels	3 days	[94]
Tombramycin/Ciprofloxacin	Skin surgery	Carbodiimide-HA	Porous mat	4 days	[95]
Various	Wound management	Dialdehyde-HA	Films	1 day	[96]

**Table 3:** Summary of cancer targeting release systems based on HA. Please see text for various abbreviations and the listed references.

Drug Type	Test Platform	Matrix System	Matrix Morphology	Release Duration	Reference
R6H4-liposome	Tumor xenograft model	HA	Coating	50 days	[102]
DOX	KB-tumor mice	Cholestryl-HA	r-GO nanocomposite	24 days	[103]
DOX	MDA-MB-231 tumor mice	Cenamide-HA	Coating	8 days	[104]
DOX	SC-C7 tumor mice	Caprolactone-HA	Nanoparticles	20 days	[105]
DOX	MDA-MB-231 tumor mice	Ceramide-HA	Nanoparticles	8 days	[108]
Paclitaxel	MCF-7 cell assay	Aminoacid-HA	Nanoparticles	4 days	[109]
Taxol	Bresat cancer HBL-100 cells	HA	Emulsion	1 day	[110]
BSA	4T1 cells	Gold-HA	Nanoparticles	14 days	[113]
Various	C4-2B cells	Hydrazide-HA	Hydrogel	1 day	[114]
DOX	MDA-MB-231 tumor mice	Ce6-HA conjugate	Nanoparticles	2 days	[115]
DOX	Nasopharyngeal CNE2 cells	Thiolated-HA	Hydrogel	3 days	[117]
DOX	(MMP)-2 cells	Poloxamer 407-HA	Nanogel	5 days	[118]
DOX	MDA-MB-231 tumor mice	rGO-HA	Colloids	18 days	[119]
Curcumin	CT26 colon cancer	Zein-HA	Nanogel	14 days	[121]
Modified coumarine	n/a	Sucrose-HA	Nanogel	6 days	[122]
